# Navigating diabetes care inequities: an observational study linking chronic care model’s structural elements to process and outcomes of type 2 diabetes care in Belgium

**DOI:** 10.1186/s12939-024-02372-4

**Published:** 2025-01-20

**Authors:** Philippe Bos, Katrien Danhieux, Edwin Wouters, Josefien van Olmen, Veerle Buffel

**Affiliations:** 1 Department of Sociology, University of Antwerp, Antwerp, Belgium; 2 Department of Family Medicine and Population Health, University of Antwerp, Antwerp, Belgium; 3 Department of Sociology, Vrije Universiteit Brussel, Brussels, Belgium

**Keywords:** Diabetes care, Chronic care model, Health inequities, Primary care, Quality of care, Belgium

## Abstract

**Background:**

Although the Chronic Care Model (CCM) provides the essential structural components of practice organisation to deliver high-quality type 2 diabetes (T2D) care, little is known about which of its elements are most important, and the extent to which it may reduce social inequities in the quality of T2D care. This study aims to assess the association between the implementation of CCM’s structural elements and the quality of T2D care processes and outcomes in Flanders (Belgium), paying specific attention to differences by patients’ socioeconomic vulnerability.

**Methods:**

We developed a longitudinal database combining information on primary care practices’ CCM implementation, with individual-level health insurance and medical lab data. Our sample included 7,593 T2D patients aged 40 years and above from 58 primary care practices in Flanders, followed up from 2017 to 2019. Medical lab data were available for a subsample of 4,549 patients. By estimating a series of hierarchical mixed-effects models, we assessed the association between primary care practices’ CCM implementation and two process and two outcome indicators of T2D care. In addition, we explored cross-level interactions with patients’ socioeconomic vulnerability.

**Results:**

Patients were more likely to have their HbA1c tested twice a year and LDL cholesterol tested yearly in practices with a higher overall CCM implementation. Regarding the different CCM elements, the clinical information system and linkages to the community were significantly associated with higher odds of being up-to-date with HbA1c testing, whereas stronger community linkages was the only dimension significantly associated with yearly LDL cholesterol testing. While socioeconomic vulnerable patients were less likely to have their HbA1c tested twice yearly, this difference disappeared in the highest-scoring practices. Regarding the outcome indicators, only a negligible proportion of variation in HbA1c and LDL cholesterol levels was due to systematic differences between practices, and hence, no clinically relevant associations with the CCM elements were found.

**Conclusion:**

Our pioneering findings support the social capital pathway, as CCM implementation is associated with a reduction in the healthcare inequity gap in the T2D care process. This suggests that promoting CCM implementation may improve healthcare equity, particularly in regions with significant socioeconomic disparities or high concentrations of deprived individuals.

**Supplementary Information:**

The online version contains supplementary material available at 10.1186/s12939-024-02372-4.

## Introduction

 Diabetes is a pervasive health concern, claiming an estimated 6.7 million lives in 2021 and accounting for 12.2% of deaths among individuals aged 20–79 years, underscoring its profound impact on public health worldwide [1]. Nearly half (45%) of the patients with diabetes are undiagnosed, pointing to the inability of healthcare systems to timely diagnose and treat people with diabetes [1]. Furthermore, inequities in diabetes care are large [3], also in high-income countries, such as Belgium [2]. These inequities are grounded in structural determinants of health but also include diabetes-specific elements such as less access to screening, high-quality care and innovations [3, 4]. This results in diabetes prevalence, morbidity, and mortality being much higher among minority groups [3]. While the general mechanisms underlying these inequities are well understood, there is less detailed empirical knowledge about how primary care practice (PCP) organisation influences the pathways between access, quality and outcomes of care.

The quality of type 2 diabetes (T2D) care is often assessed using indicators encompassing the three dimensions of Donabedian’s landmark model [5]: structure, process and outcomes. Structure comprises the fundamental components that shape the healthcare system such as accessibility, sufficient staffing, up-to-date equipment and health information systems, and a supportive policy environment. The Chronic Care Model (CCM) is a frequently used framework to assess the structural elements of care for chronic conditions, such as T2D, and identifies six essential elements to design high-quality chronic care (see Table 1). Whereas numerous adaptations to this model have been proposed, such as the Innovative Care for Chronic Conditions (ICCC) [6], the Expanded Chronic Care Model (eCCM) [7] and the Manage Care Model (MCM) [8], these alternatives are less widely adopted, lack validations and are less aligned with our research aims. This study therefore uses the CCM rather than its subsequent adaptions.

The process dimension includes aspects of the medical interaction, both at a technical and interpersonal level. It refers to the completeness, continuity, and effectiveness of activities for diagnosis, treatment and ongoing management. The outcome dimension, on the other hand, covers both intermediate and long-term health outcomes. For T2D, the frequency of glycated haemoglobin (HbA1c) monitoring is often used as a process quality indicator (QI) [9], whereas the proportion of patients achieving glycaemic control serves as an outcome-related QI [10].

**Table 1:** Elements of the chronic care model

Health Organisation	Chronic illness management programmes can be more effective if the overall system in which care is provided is oriented and led in a manner that allows for a focus on chronic illness care.
Community Linkages	Linkages between the health system and community resources play important roles in chronic illness management.
Self-management Support	Effective self-management support can help patients and families cope with the challenges of living with and treating chronic illnesses and reduce complications and symptoms.
Decision Support	Effective chronic illness management programmes ensure that providers have access to evidence-based information necessary to care for patients. This includes practice guidelines or protocols, specialty consultation, provider education and activating patients to make provider teams aware of effective therapies.
Delivery System Design	Evidence suggests that effective chronic illness management involves more than simply adding additional interventions to a current system focused on acute care. It may necessitate changes to the organisation of practice that impact the provision of care.
Clinical Information Systems	Timely, useful information about individual patients and populations of patients with chronic conditions is a critical feature of effective programmes, especially those that employ population-based approaches.

Since the origin and implementation of the CCM across the world, multiple studies have tested its effectiveness in the field of diabetes and beyond. Systematic reviews [11–14] and meta-analyses [15–17], mostly entailing (randomized) trials, demonstrate a small positive effect of structural interventions implementing (elements of) the CCM on HbA1c control. The impact on other outcomes, such as cholesterol levels, is less clear [16]. In contrast to Donabedian’s outcome dimension, the process dimension is usually not assessed, especially not in trial settings. In trials, processes such as periodic measurements of HbA1c are part of the design [18] and can, therefore, not be used as a QI.

In addition, most studies examining the impact of the CCM on diabetes outcomes do not disentangle the overall effect of the CCM into its individual components. While implementing more than two CCM elements has been shown to yield a greater effect on outcomes [15], it is unclear which elements are most effective, if any [13]. One study found that community linkages and delivery system design were associated with better cardiovascular risk outcomes [19]. Although the element clinical information systems showed a positive association with diabetes outcome QIs in a Dutch study [20], it was negatively associated in another study [19]. System-targeted initiatives proved more effective in reducing cardiometabolic outcomes than strategies solely targeting health care providers [21]. It is therefore of significant interest to explore the nuances of the CCM to determine whether a specific element serves as the linchpin in diabetes care, or if the synergy among multiple components is more important.

The extent to which the implementation of the CCM addresses inequities in the T2D care process and outcomes is still a topic of debate. The equity gap in healthcare can be explained by two distinct theories [22]: the materialist perspective highlights structural and resource-related factors such as education, income, and access to care, directly shaping health outcomes. For example, income influences access to essential resources such as healthcare and housing. In contrast, the social capital interpretation underscores psychosocial influences, such as social cohesion, trust, and discrimination, contributing to health disparities independent of material conditions. Trust in healthcare providers and social networks, as well as perceptions of inequality, play pivotal roles in shaping health outcomes. The different lenses lead to distinct policy consequences. The materialist perspective advocates addressing material deprivation, while the social capital interpretation suggests policies addressing both material and psychosocial needs to mitigate healthcare inequalities.

Following the social capital theory, it can be hypothesized that the CCM can mitigate social inequities as people at greatest risk for ill-health, often overrepresented in disadvantaged socioeconomic groups, could benefit most from the CCM. One can argue that the CCM can contribute to the social capital of the community and patients, through raising patients’ self-efficacy, improving self-management skills, stimulating collaborative care and improving communication and relationships between patients and HCWs. This, in turn, may also increase the social capital of the community, enhancing social cohesion and collective trust in the healthcare system. As those with a lower socioeconomic status tend to have lower self-efficacy [23–25], poorer communication skills, are often less integrated in the community [26] and have less trust [27] in the healthcare system, the potential impact of the CCM through social capital will be the strongest among them.

In contrast, there are also reasons to believe that the CCM will not have a health equity effect, as it does not influence the structural and material causes of social inequalities in health and health care, which are the drivers according to the materialist pathway [28, 29]. This may hold especially true in the absence of other social policies devoted to mitigating the impact of peoples’ socioeconomic status on health [30]. These theoretical effects that the CCM may (or may not) have on inequities in T2D care, have however, — to the best of our knowledge — not yet been empirically investigated.

In this study, we aim to address the abovementioned gaps by examining the link between structural elements, processes and health outcomes of T2D care, and the effects the implementation of the CCM may have on equity in T2D care in Flanders (Belgium). In Belgium, slightly more than one in three (37%) patients with diabetes are unaware of their disease and 19% of the patients using diabetes medication are not well-controlled [31]. The country’s healthcare system has substantial inequities in access to and quality of care [4]. The disparity in unmet medical needs surpasses the average of all Western European countries, with 7% among the poorest and 0.1% among the wealthiest quintiles [32]. People with lower education are 3.6 times more likely to suffer from ignored or poorly controlled diabetes [2].

This study has three objectives. First, we study the impact of the level of implementation of the CCM in PCPs on both process and outcome QIs of T2D care. We hypothesise that practices with a higher degree of CCM implementation will demonstrate higher scores on the process and outcome QIs. Furthermore, we expect a more pronounced impact of the CCM on the process QIs, given their stronger susceptibility to structural and organisational influences as compared to the outcome QIs. Second, we measure and compare the impact of the overall CCM implementation and its separate elements, on both process and outcome QIs, to evaluate which CCM elements are most influential. Finally, we examine whether the impact of CCM implementation on T2D care differs between socioeconomic vulnerable and non-vulnerable patients. Our study will be the first to test the health equity effect of CCM implementation among T2D patients.

## Data and method

### Study design and setting

The compulsory Belgium health insurance covers 99% of the population for a wide range of services, albeit with considerable co-payments. Patients have free choice of provider, and there is no gatekeeping function, so patients can visit multiple general practitioners (GPs) and have direct access to specialist care. However, financial policies, such as the Global Medical Record (GMR), are used to channel patient behaviour according to the gatekeeper model [33]. Patients who opt in for the GMR allow a GP to manage their medical information and will have lower co-payments.

PCPs are either financed through a fee-for-service (FFS) model (in 2018, serving 94% of all patients) or a capitation system [34]. A growing but still modest proportion of 41% of GPs work in a group practice; only 30% work in a multidisciplinary setting [35]. Capitation practices typically have nurses and potentially also other care providers, such as dieticians, physiotherapists or psychologists in their team; in fee-for-service practices, this is uncommon, as a remuneration system for care by nurses is lacking [36].

### Data sources

This study draws on a longitudinal database combining information on the structure, process and outcome QIs of T2D care in three regions in Flanders (Belgium): the urban regions Antwerp and Ghent and the semirural region the Campine. The study population consisted of a retrospective cohort of T2D patients aged 40 years and over in 2017 who were treated in a PCP located in one of these three regions. Because Belgium does not have a national diabetes registry [37] or a centrally managed and standardized electronic health record system, nor a systematic recording of structure-related QIs [38], the database was developed from the ground up by combing (a) self-collected data by the research team on structural indicators of T2D care at the level of PCPs with (b) individual-level health insurance and (c) medical lab data on the process and outcome indicators of T2D care, respectively (see Table 2). As the development of the database is covered in great detail elsewhere [38], only the main points are briefly discussed below.

**Table 2:** Data sources used to measure different dimensions of quality of T2D care

Dimension of quality of care	Level	Data source	Period	sample size
Structure	primary care practice	self-collected by the researchers	2019	58
Process	patient	health insurance data (IMA)^a^	2017-19	7,645
Outcomes	patient	lab data	2017-19	4,565

^a^If process data were incomplete in the health insurance database, lab data were used

This study employed a disproportionate stratified single-stage cluster sampling design with PCPs as the primary sampling units (PSUs) and T2D patients as the secondary sampling units (SSUs). The sampling frame for the PSUs consisted of all practices operational in one of the three study regions in 2019. Based on publicly available lists, the practices were stratified based on region and practice type. PCPs were subsequently randomly drawn and invited to participate in the study until a predefined sample size in each of the strata was reached. A total of 66 PCPs agreed to participate [38, 39]; however, eight were ineligible as they did not contain any SSUs of interest. Hence, the sampling of PSUs resulted in a final sample of 58 PCPs, with a response rate of 26.1%.

Within each PSU, all T2D patients aged 40 years and above in 2017 were selected as SSUs and retrospectively followed up on a yearly basis until 2019. The selection of the T2D patient population of the participating practices was performed using the national database of the Intermutualistic Agency (IMA) [40]. IMA is a joint venture that combines data from the seven national sickness funds collectively managing the Belgian compulsory health insurance. The IMA database contains reimbursement claims data for all (para)medical interventions, medications dispensed in public pharmacies and limited socio-demographic characteristics. As the IMA database does not include diagnostic data, patients were algorithmically identified as having T2D based on the proxies of taking T2D medication or registration in a pre-trajectory for T2D [38]. Patients using an insulin pump were excluded, in order to restrict the cohort mainly to patients with T2D. This resulted in an identified cohort of 7,645 T2D patients from 58 PCPs.

Finally, we additionally collated data on our study cohort’s lab test results. As medical lab data in Belgium are not centralized but distributed among a multitude of recognized laboratories [38], we compiled a list of twelve laboratories that may have captured clinical data of our sample of T2D patients. These labs were identified by asking the GPs of the participating PCPs for the names of all labs with which they cooperate. Additionally, we included the labs of the hospitals in the study regions, as T2D patients might be referred to these labs by a specialist or during a hospital stay. Eight of the twelve labs contacted agreed to participate in our study, providing data on lab test results of 4,565 (59.7%) T2D patients in our sample [38].

A common unique identifier allowed the linkage of T2D patient characteristics with lab test outcomes and primary care characteristics. After removing observations with incomplete information on at least one of the variables used in the analysis (1.3%), two analytic samples were obtained: the full IMA sample, with 7,593 T2D patients from 58 practices contributing a total of 21,939 person-years to the analysis and 2) the subsample for whom we have lab test data available, with 4,549 T2D patients from 58 practices, contributing a total of 13,251 person-years.

### Measures

#### Process and intermediate outcomes of care

In this study, four dependent variables measure the quality of T2D care, related to HbA1c and low-density lipoprotein-cholesterol (LDL-C) ─ one process and one outcome indicator for each. HbA1c and LDL-C were selected as clinical indicators due to their strong correlation with health outcomes and their inclusion in international diabetes care guidelines [41, 42]. Optimal HbA1c and LDL-C levels reduce the risk of diabetes complications and cardiovascular disease [41, 42], providing a comprehensive assessment of care quality.

The HbA1c process QI is a dichotomous variable measuring whether at least two HbA1c tests were recorded for a given patient in the past 12 months, spaced at least 80 days apart. This 80-day cut-off was chosen as HbA1c reflects glycaemic control over a period of 3 months. Multiple HbA1c tests within this timeframe are thus uninformative and considered overuse [43]. The dichotomous LDL-C process QI measures whether, in the past 12 months, at least one LDL-C test ─ either measured or calculated ─ was recorded [41]. Finally, the HbA1c and LDL-C outcome QIs are continuous variables reflecting the average HbA1c value in % and the average LDL-C value in mg/dl, respectively, of all observed tests for a given patient in a single year.

#### Structure of care

To measure the degree of implementation of the CCM in the PCPs, we used the Assessment of Chronic Illness Care (ACIC), a comprehensive tool designed to evaluate the delivery of care for chronic illness along the six CCM dimensions (cf. Table 1) [44]. It has previously been used to measure the quality of T2D care in Belgium [39, 45] and has been validated in the Netherlands [46]. The data on the ACIC scores of the participating PCPs were collected during a previous study [39]. Briefly put, an interview guide with open-ended questions was developed based on the ACIC questionnaire. Two researchers visited each participating PCP and interviewed the healthcare practitioners about their practice’s organization. Based on these interviews, the researchers completed the ACIC questionnaire, resulting in a subscale score for each CCM dimension and a total ACIC score, calculated as the average across all six subscale scores. A more detailed elaboration on the collection of ACIC scores is provided elsewhere [38, 39]. All ACIC scores were grand mean centered for the analysis.

#### Socioeconomic vulnerability

To identify socioeconomic vulnerable patients, we included a time-varying variable indicating whether the patient was entitled to *increased reimbursement* (IR) for healthcare expenditure (1/0). The system of IR is the predominant social safety net in Belgium for healthcare expenses, providing various benefits, such as reduced co-payments for healthcare services and a third-party payer system for visits to the GP, wherein only the co-payment needs to be paid at the point of care rather than the full fee [47]. Eligibility for IR is either granted automatically based on the uptake of other social benefits or by passing a household income test [47]. As such, IR status is often used as a proxy for socioeconomic vulnerability in studies using Belgian administrative data [e.g. [48] ─ as is the case in the current study.

#### Control variables

Finally, we also included a number of control variables at both the practice and individual level. First, we included *practice type* as a categorical predictor distinguishing between three categories: monodisciplinary FFS [ref.], multidisciplinary FFS and multidisciplinary capitation-based. Next, a set of individual-level covariates that capture socio-demographic characteristics, health status and healthcare utilization patterns of the cohort of T2D patients was included to control for compositional differences in the patient population of PCPs. The socio-demographic variables include: *age at baseline* categorized into three groups (40–59 [ref.], 60–79 and 80 + years); *gender* (female [ref.], male); an indicator of whether one *lived alone* (1/0); and whether one *died* in the respective year (1/0). *Observation year* (2017 [ref.], 2018, 2019) was included to capture time trends. As regards the health and healthcare use characteristics, we included the *number of comorbidities* and the *number of GPs* contacted during a calendar year. The former is a continuous variable measuring the number of co-occurring chronic diseases or disease groups (out of a total of 19) that patients have in addition to T2D (see annex text A.1 for the list of chronic conditions and how these were identified). The latter was included as a continuous variable centered on 1, measuring the number of different GPs a patient contacted during a year.

### Analysis

**Fig. 1:**
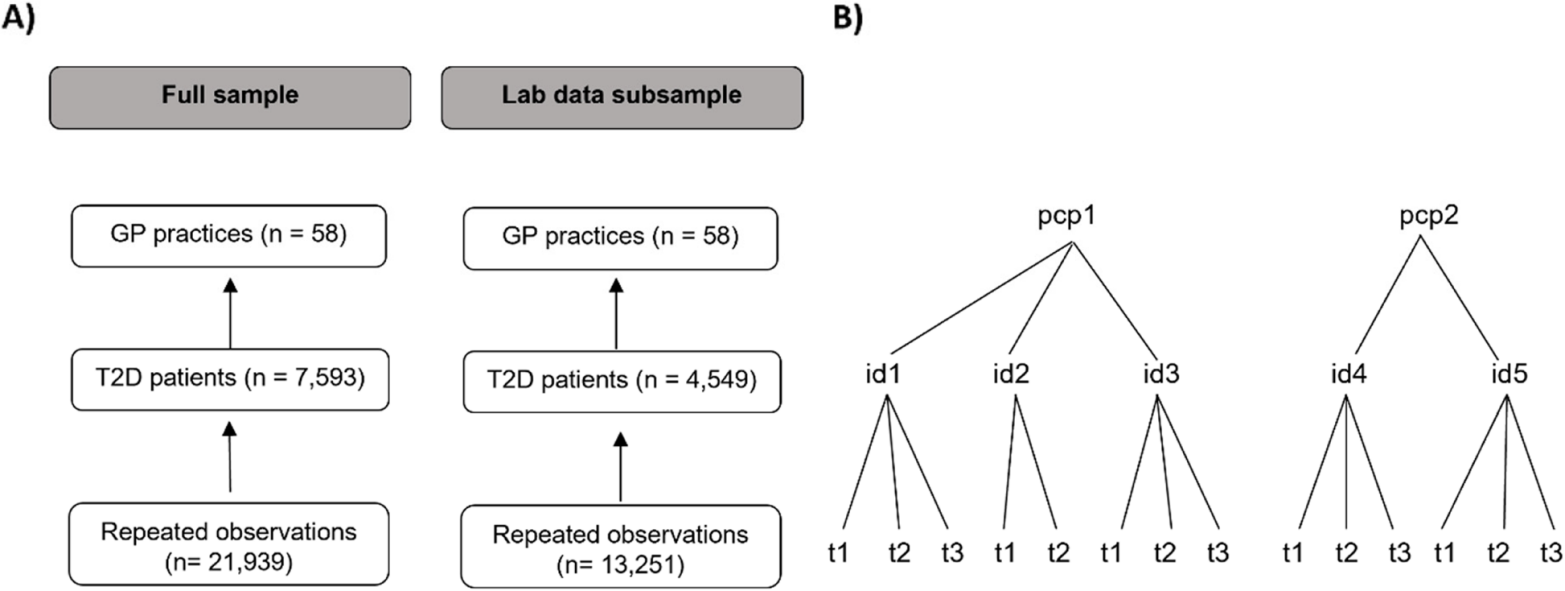
**(A)** Unit diagram for the full sample and lab data subsample and **(B)** classification diagram with repeated observations nested within T2D patients, nested within PCPs

To account for the hierarchically clustered nature of the data, and to assess the contextual effect of the CCM on the process and outcome QIs, we estimate a series of hierarchical mixed-effects models for each of the dependent variables [49]. The models have a nested three-level structure: repeated measurements nested within patients, in turn, nested within PCPs. See Fig. 1 for the unit and classification diagram depicting the nested structure for both analytic samples. Hierarchical mixed models are well-suited for the current analysis as they assume that the higher-level units are a random sample of PCPs, allowing statistical inference to the larger population of PCPs within the study region.

The dichotomous process QIs were analysed using generalized linear mixed-effects models (GLMMs) with a logit link. For the HbA1c process indicator, all T2D patients for whom IMA data was available (*n* = 7593) were included in the analysis. As LDL-C is often calculated from other lipid profile components rather than directly measured, not all LDL-C measurements were recorded in the IMA database. We therefore relied on the data provided by the labs to assess whether an LDL-C test (either measured or calculated) was performed; hence, only the subsample for whom lab data is available (*n* = 4549) was included in the analysis for this process QI.

The continuous outcome QIs were analysed using linear mixed-effects models (LMMs). Only patients of the lab data subsample for whom at least one HbA1c and/or LDL-C test were available were included in the analysis. For the HbA1c outcome, this amounted to 4394 T2D patients (96.6% of the lab data subsample) and for the LDL-C outcome, to 4192 patients (95.4% of the lab data subsample).

The analytic strategy is identical for each of the dependent variables. First, to assess the association between the implementation of the CCM and the process and outcome QIs, a set of eight models is fitted for each dependent variable. We started by estimating a reference model controlling for: (a) patient characteristics to account for compositional differences in the patient population between PCPs; and (b) practice type, as this turned out to be an important confounder of the relationship of interest. The selection of covariates was performed using a backward stepwise procedure. The total ACIC and subscale scores were subsequently separately added as predictors to the reference model, to assess their impact on the process and outcome QIs. The relationships between the ACIC (subscale) scores and the continuous outcome QIs, and the log-odds of the dichotomous process QIs, were constrained to be linear, as alternative non-linear specifications did not yield improvements in model fit.

In addition to examining the fixed coefficients, we examine the variance components of the models to grasp the relevance of CCM implementation in accounting for between-practice variation in the process and outcome QIs. Specifically, for each model, we calculated: (a) the intraclass correlation coefficient for the practice level ($\:{\text{I}\text{C}\text{C}}_{\text{p}}$) and (b) the proportional change in practice-level variance ($\:{\text{P}\text{C}\text{V}}_{\text{p}}$) relative to the reference model.

The $\:{\text{I}\text{C}\text{C}}_{\text{p}}$ reflects the proportion of the total unexplained variation in the outcome attributed to systematic differences between PCPs [50]. It is calculated as

$$\:{{ICC}_{p}}_{=}\:\frac{{\sigma^{2}}_{p}}{{{\sigma^{2}}_{p}+\sigma^{2}}_{i}+{\sigma^{2}}_{e}}*100$$

where $\:{\sigma^{2}}_{p}$ is the unexplained variance in the process and outcome QIs between practices, $\:{\sigma^{2}}_{i}$ captures the variance attributed to differences between patients within practices, and $\:{\sigma^{2}}_{e}$ is the residual variance term capturing variation in T2D care within individuals over time. In contrast to LMMs, GLMMs do not estimate a residual variance term $\:{\sigma^{2}}_{e}$. As such, we relied on the latent variable approach for estimating the$\:\:{\text{I}\text{C}\text{C}}_{\text{p}}$ for the process indicators, by assuming that $\:{\sigma^{2}}_{e}=\frac{\:{\pi\:}^{2}}{3}$ [49, 50].

The $\:{\text{P}\text{C}\text{V}}_{\text{p}}$ represents the proportional change in between-practice variance as a result of including one of the ACIC (subscale) scores, relative to the reference model [50]. A decrease in between-practice level variance indicates that the respective ACIC score is relevant in explaining variation in process and outcome QIs between practices. It is calculated as

$$\:{{PCV}_{p}}_{=}\:\frac{{\sigma^{2}}_{p1}-{\sigma^{2}}_{p2}}{{\sigma^{2}}_{p1}}*100$$

where $\:{\sigma^{2}}_{p1}$ and $\:{\sigma^{2}}_{p2}$ are practice-level variances of respectively the reference model and the model with one of the ACIC scores as an additional term.

Uncertainty in the estimates for the between-practice variance $\:{\sigma^{2}}_{p}$, the $\:{\text{P}\text{C}\text{V}}_{\text{p}}$ and the $\:{\text{I}\text{C}\text{C}}_{\text{p}}$ is obtained by applying a non-parametric bootstrap procedure [51, 52]. We drew 2000 bootstrap samples in which we randomly selected 58 PCPs with replacement, and in turn, all of their patients and repeated observations. The abovementioned models were refitted to each bootstrap sample and 95% confidence intervals (CIs) of the variance measures were determined using the 2.5 and 97.5% percentiles.

Finally, to assess whether the relationships between the ACIC (subscale) scores and the process and outcome QIs differ according to socioeconomic vulnerability, a set of eight models was fitted for each dependent variable. First, a random coefficient model was estimated to assess the relationship between IR status and the process and outcome QIs and test its random slope variance. Subsequently, each ACIC score was separately added as a predictor to the model, including a cross-level interaction with IR status, to assess its impact on the QIs.

All analyses were performed in R version (4.3.0) [53]. The mixed-effects models were estimated using the lme4 package [54], assuming that the higher-level random effects are drawn from a normal distribution. Sampling weights were calculated and used in all analyses to account for the unequal selection probabilities and to compensate for non-response at the level of the practices [55]. The calculation of these weights is detailed in annex text A.2.

## Results

### Descriptive results

The weighted descriptive statistics of all variables are provided in annex Table A.1. For 68.5% of the person-years of the selected T2D patients, HbA1c was measured at least twice yearly and for 75.3% of the person-years of the lab data subsample, at least one LDL-C test was recorded. The average HbA1c was 7.04% (SD = 1.04) and the average LDL-C amounted to 82.44 mg/dl (SD = 31.00). On average, the T2D patients in our study region had their regular GP working in a PCP that provided only basic support for chronic illness care, with an overall ACIC score of 3.65 (SD = 1.16) on a scale of 11. The different ACIC subscale scores ranged, on average, between 2.38 (SD = 1.52) for the community linkages subscale and 5.99 (SD = 1.55) for the healthcare organisation subscale.

### Regression analysis

#### Process of care

Table 3 summarises the GLMMs assessing the relationship between the ACIC scores and both process indicators (see annex Tables A.2 and A.3 for all model estimates). The variance-decomposition in the null model shows substantial variation in both process QIs between PCP practices. For the HbA1c process QI, 16.40% (95% CI: 10.98–22.28) of the variation was due to systematic differences between practices, whereas 67.88% (95% CI: 61.99–73.48) of the variation was between individuals within practices. For the LDL-C process QI, these figures are respectively 28.57% (95% CI: 20.99–35.02) and 51.45% (95% CI: 43.56–59). The remaining variation in both process QIs reflects changes within individuals over time. The variance components of the reference models show that, when controlling for compositional differences in terms of patient population and practice type, the proportions of unexplained variation between PCPs remain almost unchanged as compared to the null model for the HbA1c QI (ICC_p_: 16.87%, 95% CI: 9.99–23.35) and reduces slightly to 24.85% (95% CI: 15.94–31.35) for the LDL-C QI. Hence, a substantial contextual effect remains to be explained.

The fixed effects results indicate a significant positive association between the total ACIC score and both process QIs. Controlling for compositional differences and practice type, the odds of having HbA1c tested twice a year and LDL-C yearly, more than doubled for each one-unit increase in the total ACIC score (respectively AOR: 2.17, 95% CI: 1.22–3.84 and AOR: 2.07, 95% CI: 1.06–4.03). In addition, the variance components indicate that the total ACIC score explains 21.23% (95% CI: 0.08–53.64) and 13.60% (95% CI: 0.95–37.41) of the variation between PCPs in the HbA1C and LDL-C process QI, respectively.

As regards the different elements of the CCM, we see that stronger linkages to the community (AOR: 1.59, 95% CI: 1.14–2.24) and a higher score for the clinical information system (AOR: 1.39, 95% CI: 1.02; 1.90) were significantly associated with higher odds of having HbA1c tested twice a year. Both elements also explain a significant amount of variation in the HbA1c QI between practices ─ respectively 23.14% (95% CI: 0.07–55.86) and 14.39% (95% CI: 0.03.; 49.33). In contrast, stronger community linkages was the only dimension of the CCM significantly associated with higher odds of receiving a yearly LDL-C test (AOR: 1.75, 95% CI: 1.21–2.51), which accounts for 24.19% (95% CI: 6.38–48.87) of the inter-practice variation.

**Table 3:** Results of the GLMMs for the association between ACIC (subscale) score(s) and the process indicators

	Fixed effects		Variance components	
Model	AOR [95% CI]	sig.	PCV_*p*,_ % [95% CI]	ICC_*p*_, % [95% CI]
**HbA1c process indicator (0/1)**				
Null model^a^				16.40 [10.98─22.28]
Reference model^b^			Ref.	16.87 [9.99─23.35]
+Total ACIC	2.17 [1.22—3.84]	**	21.23 [0.08—53.64]	13.78 [7.7—19.55]
+Health Organisation	1.08 [0.77—1.52]		0.74 [−34.07—23.47]	16.76 [9.78—23.21]
+Community linkages	1.59 [1.14—2.24]	**	23.14 [0.07 55.86]	13.49 [7.23—19.31]
+Self-management support	1.39 [0.89—2.16]		6.70 [−28.59—33.1]	15.92 [9.37—22.33]
+Decision support	1.44 [0.80—2.59]		5.69 [−20.43—39.17]	16.06 [8.57—22.75]
+Delivery System design	1.50 [0.97—2.32]		11.03 [−13.59—44.09]	15.29 [8.25—21.64]
+Clinical information system	1.39 [1.02—1.90]	*	14.39 [0.03—49.33]	14.80 [8.32—21.32]
**LDL-C process indicator (0/1)**				
Null model^a^				28.57 [20.99—35.02]
Reference model^b^			Ref.	24.85 [15.94—31.35]
+Total ACIC score	2.07 [1.06— 4.03]	*	13.60 [0.95—37.41]	22.23 [13.27—28.26]
+Health Organisation	1.39 [0.96—2.02]		5.40 [−0.95—20.86]	23.84 [15.22—29.86]
+Community linkages	1.75 [1.21—2.51]	**	24.19 [6.38—48.87]	20.05 [12.03—25.01]
+Self-management support	1.35 [0.82—2.22]		4.48 [−0.08—22.49]	24.01 [14.82—30.27]
+Decision support	1.27 [0.67—2.42]		2.37 [−0.13—22.04]	24.41 [14.73—30.69]
+Delivery System design	0.96 [0.59—1.58]		−0.02 [−0.13—18.8]	24.86 [15.37—30.66]
+Clinical information system	1.21 [0.85—1.73]		5.02 [−0.3—25.89]	23.90 [14.52—30.03]

***p ≤ 0.001; **p ≤ 0.01; *p ≤ 0.05

*AOR* adjusted odds ratio, *CI* confidence interval, *PCV*_p_ proportional change in between-practice variance as compared to the reference model, *ICC*_p_ intraclass correlation coefficient for the practice level

^a^Random intercept model without predictors

^b^Random intercept model adjusting for the individual and practice-level control variables

The main results of the models assessing the differential relationship between the ACIC (subscale) scores and the process QIs are summarized in Table 4 (see annex Tables A.6 and A.7 for all model estimates). The random slope model indicates that, controlling for patient characteristics and practice type, beneficiaries of IR, were, on average, significantly less likely to have their HbA1c tested at least twice a year than non-beneficiaries (AOR: 0.65, 95% CI: 0.44–0.96). In contrast, there were no significant differences in the odds of having a yearly LDL-C measurement between both groups. Moreover, the addition of the random effect for IR status led to a significant model improvement for both the HbA1c process indicator (∆−2LL: 138.16; ∆df.: 2; *p* < 0.001) and the LDL-C process indicator (∆−2LL: 237.35; ∆df.: 2; *p* < 0.001), indicating that there is substantial variation in the respective relationships between PCPs.

**Table 4:** Results of the GLMMs showing the cross-level interactions between increased reimbursement status and each of the ACIC scores on the process indicators

	increased reimbursement		ACIC (subscale) score		Interaction	
**Model**	AOR [95% CI]	sig.	AOR [95% CI]	sig.	AOR [95% CI]	sig.
**HbA1c process indicator (0/1)**						
Random slope model^a^	0.65 [0.44—0.96]	*				
Cross-level interactions models^b^						
Total ACIC score	0.71 [0.49—1.02]		1.77 [0.98—3.22]		1.36 [1.05—1.76]	*
Health Organisation	0.63 [0.38—1.05]		1.03 [0.57—1.85]		1.07 [0.77—1.49]	
Community linkages	0.67 [0.46—0.97]	*	1.46 [1.02—2.09]	*	1.16 [0.93—1.43]	
Self-management support	0.71 [0.49—1.03]		1.21 [0.76—1.91]		1.25 [1.02—1.52]	*
Decision support	0.69 [0.48—0.99]	*	1.16 [0.62—2.17]		1.54 [1.11—2.15]	*
Delivery System design	0.75 [0.53—1.07]		1.30 [0.83—2.04]		1.26 [1.08—1.47]	**
Clinical information system	0.67 [0.39—1.14]		1.26 [0.94—2.04]		1.14 [0.88—1.46]	
**LDL-C process indicator (0/1)**						
Random slope model^a^	1.26 [0.77—2.05]					
Cross-level interactions models^b^						
Total ACIC score	1.14 [ 0.70—1.85]		2.35 [1.18—4.69]	*	0.77 [0.56—1.08]	
Health Organisation	1.22 [0.75—1.99]		1.48 [0.99—2.20]		0.85 [0.61—1.17]	
Community linkages	1.21 [0.74—1.97]		1.85 [1.26—2.72]	**	0.88 [0.68—1.16]	
Self-management support	1.18 [0.72—1.93]		1.46 [0.87—2.45]		0.85 [0.65—1.12]	
Decision support	1.23 [0.77—1.98]		1.40 [0.71—2.77]		0.75 [0.49—1.17]	
Delivery System design	1.16 [0.71—1.89]		1.03 [0.62—1.73]		0.86 [0.69—1.06]	
Clinical information system	1.14 [0.70—1.85]		1.29 [0.89—1.87]		0.86 [0.70—1.07]	

***p ≤ 0.001; **p ≤ 0.01; *p ≤ 0.05; *AOR* adjusted odds ratio, *CI* confidence interval

^a^Random slope model including a random effect for increased reimbursement status, while adjusting for the individual and practice-level control variables

^b^Random slope models including a random effect for increased reimbursement status and its cross-level interaction with one of the ACIC (subscale) scores, while adjusting for the individual and practice-level control variables

We see that the associations between the HbA1c process indicator and four of the ACIC scores differ significantly between beneficiaries of IR and non-beneficiaries, with the former benefitting more from the implementation of the respective CCM elements than the latter. The increase in odds of having HbA1c measured at least twice a year for each one-unit increase in the total ACIC score (AOR: 1.36, 95% CI: 1.05–1.76) as well as in the subscale scores for self-management support (AOR: 1.25, 95% CI: 1.02–1.52), decision support (AOR: 1.54, 95% CI: 1.11–2.15), and delivery system design (AOR: 1.26, 95% CI: 1.08–1.47), was significantly greater among those entitled to IR than those who were not.

**Fig. 2:**
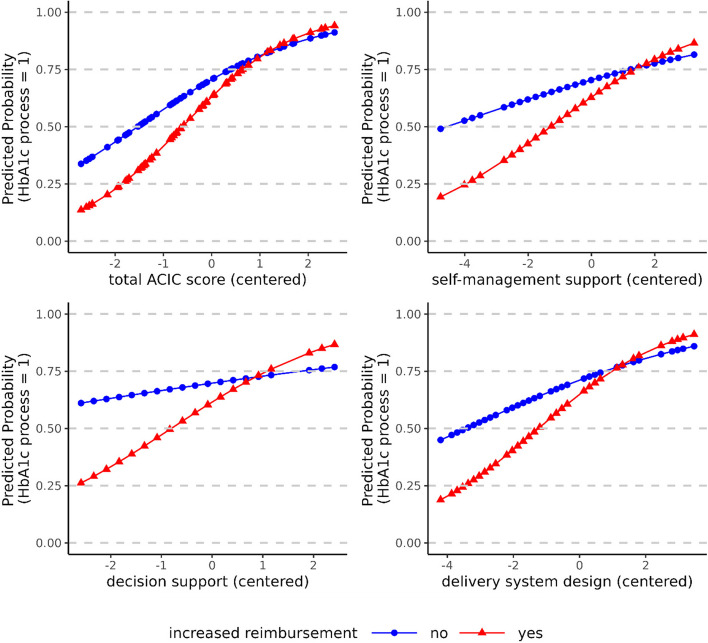
Interaction plots of the significantly different relationships between the ACIC (subscale) scores and the HbA1c process indicator by increased reimbursement status. Notes: The plot shows the estimated marginal means for the HbA1c process indicator for the significant (*p* < 0.05) interaction effects between the ACIC (subscale) scores and IR status. The marginal means are plotted over the range of observed values for each ACIC (subscale) score and calculated from the GLMMs testing the respective cross-level interactions

For ease of interpretation, we provide graphical illustrations of the significant interaction effects (Fig. 2). These demonstrate that T2D patients with IR were, on average, less likely to be up-to-date with respect to HbA1c screening in practices with low to average values for the total ACIC score and the self-management support, decision support and delivery system design subscale scores. However, as the estimated slope for these ACIC (sub)scores is steeper among those with IR than among non-beneficiaries, the difference between both becomes negligible (or even reverses) for practices with the highest levels of implementation of the CCM. In contrast to the HbA1c process QI, we observe no significant differences by IR status in the relationship between any of the ACIC scores and the LDL-C process QI.

#### Outcomes of care

The results of the LMMS assessing the relationship between the ACIC scores and each of the outcome QIs are summarized in Table 5 (see annex Tables A.4 and A.5 for all model estimates). The variance-decomposition of the null model indicates that the observed variation in both outcomes was attributed almost entirely to changes within individuals over time and between individuals. For HbA1c, this amounts to respectively 63.53% (95% CI: 53.56–72.42) and 33.94% (95% CI: 25.90–43.65) of the total variation. The proportions for LDL-C were similar, accounting respectively for 66.59% (95% CI: 58.36–73.80) and 32.23% (95% CI: 25.20–40.31) of the total variation. Only a negligible proportion of the variation in HbA1c and LDL-C can be attributed to differences between practices, respectively 2.52% (95% CI: 0.76–4.46) and 1.18% (95% CI: 0.45–1.99). When controlling for individual covariates and practice type in the reference model, the between-practice variation in both outcomes decreases further. Only 1.19% (95% CI: 0.27–2.03) of the variation in HbA1c not yet accounted for by the model was attributed to differences between practices, whereas the unexplained between-practice variation in LDL-C was no longer significantly different from 0.

Despite the non-significant between-practice variation, we see that patients’ LDL-C values were significantly lower in practices with a higher total ACIC score (B=−2.20, *p* ≤ 0.05), and self-management support (B=−1.43, *p* ≤ 0.05) and delivery system design subscale scores (B=−0.78, *p* ≤ 0.05). The reduction in between-practice variation in LDL-C as a result of controlling for these scores is, however, not significantly different from 0, as there was no significant between-practice variation to begin with. In contrast to the LDL-C outcomes, none of the ACIC scores were significantly related to patients’ HbA1c levels.

**Table 5:** Results of LMMs on the association between ACIC (subscale) score(s) and yearly average HbA1c and LDL-C levels

	Fixed effects		Random effects	
Model	B (SE)	sig.	PCV_*p*_ [95% CI]	ICC_*p*_ [95% CI]
**HbA1c value (%)**				
Null model^a^				2.52 [0.76—4.46]
Reference model^b^			Ref.	1.19 [0.27—2.03]
+Total ACIC score	−0.01 (0.04)		−4.69 [−12.96—13.69]	1.25 [0.27—2.09]
+Health Organisation	−0.01 (0.02)		−4.95 [−16.38—16.08]	1.25 [0.24—2.09]
+Community linkages	−0.00 (0.02)		−5.11 [−15.87—21.06]	1.25 [0.29—2.11]
+Self-management support	−0.01 (0.03)		−3.29 [−11.18—21.29]	1.23 [0.26—2.07]
+Decision support	0.00 (0.04)		−4.11 [−14.02—20.71]	1.24 [0.26—2.1]
+Delivery System design	0.01 (0.03)		−4.83 [−12.38—16.76]	1.25 [0.25—2.08]
+Clinical information system	−0.02 (0.02)		−1.44 [−9.69—28.76]	1.21 [0.23—2.02]
**LDL-C value (mg/dl)**				
Null model^a^				1.18 [0.45—1.99]
Reference model^b^			Ref.	0.53 [0—1.3]
+Total ACIC score	−2.20 (0.94)	*	10.98 [−54.36—100]	0.47 [0—1.1]
+Health Organisation	0.13 (0.56)		−6.98 [−52.82—90.99]	0.57 [0—1.33]
+Community linkages	−0.75 (0.52)		−3.65 [−68.3—45.38]	0.55 [0—1.29]
+Self-management support	−1.43 (0.71)	*	17.15 [−17.36—100]	0.44 [0—1.12]
+Decision support	−1.10 (0.85)		−7.32 [−132.58—24.37]	0.57 [0—1.31]
+Delivery System design	−1.33 (0.60)	*	11.34 [−72.89—100]	0.47 [0—1.09]
+Clinical information system	−0.78 (0.43)		−3.85 [−141.76—51.42]	0.55 [0—1.24]

***p ≤ 0.001; **p ≤ 0.01; *p ≤ 0.05; *SE* standard error, *CI* confidence interval, *PCV*_p_ proportional change in between-practice variance as compared to the reference model, *ICC*_p_ intraclass correlation coefficient for the practice level

^a^Random intercept model without predictors

^b^Random intercept model adjusting for the individual and practice-level control variables

The results of the LMMs testing for a differential relationship between ACIC scores and both outcome QIs by socioeconomic vulnerability are summarized in Table 6 (see annex Tables A.8 and A.9 for all model estimates). The random slope model indicates that there were, on average, no significant differences between beneficiaries of IR and non-beneficiaries regarding their HbA1c, nor LDL-C levels, after adjusting for individual covariates and practice type. As the inclusion of the random effect for IR did not significantly improve model fit for both the HbA1c (∆−2LL: 1.9; ∆df.: 2; *p* = 0.39) as the LDL-C outcomes (∆−2LL: 2.25; ∆df.: 2; *p* = 0.33), there was also no systematic variation in these relationships between PCPs. Unsurprisingly then, none of the cross-level interactions of the ACIC scores with IR are significant, indicating the relationship between the ACIC scores and HbA1c and LDL-C did not differ by patients’ socioeconomic vulnerability.

**Table 6:** Results of the LMMs showing the cross-level interactions between increased reimbursement status and each of the ACIC scores on the outcome indicators

	increased reimbursement		ACIC (subscale) score		Interaction-effect	
Model	B (SE)	sig.	B (SE)	sig.	B (SE)	sig.
HbA1c value (%)						
Random slope model^a^	0.06 (0.03)					
Cross-level interactions models^b^						
Total ACIC score	0.06 (0.04)		−0.03 (0.04)		0.01 (0.03)	
Health Organisation	0.05 (0.03)		−0.02 (0.03)		0.04 (0.02)	
Community linkages	0.06 (0.03)		−0.01 (0.02)		0.01 (0.02)	
Self-management support	0.06 (0.03)		−0.02 (0.03)		0.02 (0.02)	
Decision support	0.06 (0.04)		0.00 (0.04)		−0.00 (0.03)	
Delivery System design	0.06 (0.04)		0.01 (0.03)		0.01 (0.02)	
Clinical information system	0.05 (0.04)		−0.02 (0.02)		−0.01 (0.02)	
**LDL-C value (mg/dl)**						
Random slope model^a^	−0.02 (1.12)					
Cross-level interactions models^b^						
Total ACIC score	0.28 (1.14)		−2.56 (0.96)	**	0.29 (0.83)	
Health Organisation	−0.04 (1.08)		−0.19 (0.63)		1.12 (0.76)	
Community linkages	0.07 (1.14)		−0.73 (0.56)		0.02 (0.67)	
Self-management support	0.20 (1.14)		−1.71 (0.74)	*	0.34 (0.65)	
Decision support	0.11 (1.12)		−1.37 (0.93)		0.76 (1.09)	
Delivery System design	0.11 (1.16)		−1.10 (0.42)	**	−0.14 (0.51)	
Clinical information system	0.04 (1.18)		−0.76 (0.45)		−0.07 (0.50)	

***p ≤ 0.001; **p ≤ 0.01; *p ≤ 0.05; *SE* standard error, *CI* confidence interval

^a^Random slope model including a random effect for increased reimbursement status, while adjusting for the individual and practice-level control variables

^b^Random slope models including a random effect for increased reimbursement status and its cross-level interaction with one of the ACIC (subscale) scores, while adjusting for the individual and practice-level control variables

## Discussion

In this study, we used a unique database combining health insurance data, lab data and self-collected data from PCPs, to study the impact of the overall level of implementation of the CCM and of its different elements on process and outcome QIs of T2D care. In addition, we assessed whether this impact differed between socioeconomic vulnerable and non-vulnerable patients. Our study revealed three major findings.

First, we found that CCM implementation was related to improved diabetes management: in practices with higher ACIC scores, patients were more likely to have their HbA1c measured twice a year and their LDL-C yearly. However, we found this relevant impact of the CCM only on the process QIs and not on the outcome QIs. For the HbA1c and LDL-C outcomes, our analysis showed that the observed variation was attributed almost entirely to differences between patients and changes within patients over time, rather than to systematic differences between PCPs. This suggests that individual characteristics such as nutrition, environment, lifestyle, and poverty are more important than the structural characteristics of the practices in determining the outcomes of T2D care [56].

While other studies have made attempts, only one group in Texas, USA, has effectively utilized the ACIC questionnaire as designed to evaluate the effect of the CCM in diabetes patients [19, 57, 58]. In relation to process QIs, no significant impact of the ACIC score on HbA1c or lipid measurements was observed. However, regarding the outcome QIs, patients in practices with higher total ACIC scores exhibited lower HbA1c levels in this study. They, however, used snowballing to sample the practices and recruited patients who were present at the physician’s waiting room. Both procedures increase the likelihood of bias. Especially recruiting patients in the waiting room leads to an over-selection of patients who visit the physician more often, and are therefore more likely to follow the guidelines and achieve glycaemic control. Experimental research evaluating the impact of the CCM also demonstrates an impact of the total ACIC score on HbA1c levels, as compiled in several meta-analyses [15–17].

The lack of a statistically or clinically significant association between the ACIC scores and the outcome QIs in our study may, at least in part, be attributed to selection bias. Due to the observational design, we could only include HbA1c and LDL-C test results from patients tested at least once in a given year for the respective clinical indicators. As a result, patients who did not have their HbA1c and LDL-C tested, did not contribute person-years of data to the analysis of the outcome QIs. It seems reasonable to think that those who are tested yearly receive higher quality care, and, therefore, have lower HbA1c and LDL-C levels. To the extent that this reasoning holds, the variation in the outcome QIs within our sample of patients would be limited, as the potentially worst health outcomes of patients untested during the observation window are not included in the analysis. However, this does not rule out the alternative hypothesis that healthcare has only a minor impact on health outcomes compared to other factors such as genetic predispositions, social circumstances, environmental exposures, and behavioural patterns, which collectively exert a much greater influence [59–61], as noted earlier.

The second finding is that when studying the separate CCM elements, only community linkages (for both HbA1c and LDL) and clinical information systems (for HbA1c) have a significant impact on the process QIs. This discovery holds particular significance for community linkages, as previous studies have largely overlooked this element. Notably, it is the least frequently integrated aspect in RCTs assessing the implementation of the CCM. Consequently, there are no definitive conclusions on its impact on outcomes so far [13, 14, 17, 21]. In other observational research, the relation between community linkages and process QIs was established in 2 studies [58, 62], but not considered [63–65] or not significant [66, 67] in other studies. Exploring how stronger community linkages lead to improved follow-up could be a valuable area of future research. One hypothesis is that the CCM promotes a collaborative culture in which responsibilities increase among all team members, which is described among health professionals [22]. A similar mechanism could be true for community members. In our previous study [39], we described that practices implementing the CCM referred patients more frequently to community initiatives where they could meet peers.

The association between the process QIs for diabetes and clinical information systems was observed in multiple other observational studies [62, 64, 67]. Explanations for the impact of clinical information systems on measuring HbA1c are more straightforward. Registries of patients with diabetes, reminders, and care plans all facilitate proactive and qualitative care. However, there is no clear explanation for why the other elements do not have a significant influence. Still, we can argue that the CCM should be considered as a holistic and integrated model, in which the elements are inherently linked to each other and the whole is greater than the sum of its parts. This conclusion is supported by the fact that we found evidence of a positive impact of the total ACIC score on the process QIs. Moreover, in previous studies, implementing more elements had a greater effect [15], while it was difficult to define one single most important element [68].

Third, we observed a difference in T2D management according to patients’ socioeconomic status, with lower odds of being up-to-date with HbA1c testing among those with IR for healthcare expenditure. However, this difference was smaller in PCPs with a higher CCM implementation. More concretely, the implementation of the CCM in PCPs had a stronger positive effect on the HbA1c process QI among socioeconomic disadvantaged patients. This finding provides fresh insights into the role of the CCM in improving health equity. On the one hand, our findings support the social capital pathway, arguing that the CCM will be especially beneficial for vulnerable patients through its investments in self-management support, improvement of health literacy and trust in health professionals, integration in the community and interdisciplinary working. From previous research [69, 70], we know that socioeconomic vulnerable patients have lower self-management skills, health literacy, trust in health professionals, are less socially integrated and often have complex health and social care needs, requiring interdisciplinary working.

In addition to the overall effect of the CCM, we found a stronger impact of the elements of self-management support, decision support, and delivery system design on the HbA1c process QI among socioeconomic vulnerable patients. This finding contrasts with the limited previous research, which suggests that self-management support interventions may exacerbate the social gradient [71]. However, why we observed a stronger impact of decision support, but not of community linkages, on socioeconomic vulnerable patients’ process QIs, is less understandable from a social capital perspective and strengthens again the idea that the CCM should be considered as a holistic integrated system. It can be argued that to reduce socioeconomic inequalities in health and healthcare, more structural interventions, focusing on the fundamental causes of social inequalities will be required [72]. Especially if we look at the outcome QIs, these are influenced by a wide range of social and biological factors, which cannot all be captured in the organisation of chronic care, but need a ‘health in all policies’ perspective [73].

### Strengths and limitations

Our study has several strengths, but also some limitations. The observational design has the advantage that it allows to study the associations between CCM implementation and the process QIs, whereas most previous studies have been limited to assessing intermediate outcomes of T2D care [11–17] However, this design also has its limitations, as the assumption that PCPs’ CCM implementation is exogenous may not fully hold. For instance, despite our best attempts to control for compositional differences in the patient population of PCPs, patients’ free choice of provider may still have caused selectivity unadjusted for by the model, with potentially more health-conscious patients choosing better-organized practices. Conversely, PCPs serving vulnerable populations or having poor patient outcomes may be more motivated to implement the CCM, introducing reverse causation. The limited availability of control variables and the measurement of ACIC scores at only one point in time increase the risk of omitted variable bias. Further research could improve upon this by measuring the implementation of the CCM at different points in time, allowing to establish temporal ordering and the use of fixed effects regression methods to control for any (unmeasured) time-invariant characteristics and, hence, more closely mimic the virtues of randomized experiments [74].

Next, the use of standardized insurance data collected uniformly across all practices ensures completeness, encompassing all patients and records systematically. This stands as a distinct advantage compared to medical records, which are highly dependent on accurate documentation and may reveal variations in documentation practices rather than differences in quality. However, as LDL-C levels are often not measured directly but calculated based on the other cholesterol biomarkers, not all LDL-C measurements were recorded in the health insurance data. As a result, we relied on lab data to determine whether an LDL-C test had been performed. Unfortunately, this strategy is not flawless and bears the risk of misclassification: it is possible that, although we did not observe an LDL-C measurement in the lab data for a given patient, one had nevertheless been performed by another lab that did not participate in our study.

Furthermore, although our study is among the first to study heterogeneity in the association between CCM implementation and quality of T2D care, we relied on a rather broad proxy to identify socioeconomic vulnerable patients — that is, whether the patient receives IR for healthcare expenditure. As there is significant non-take-up of this benefit despite meeting the eligibility criteria [47], it is likely that not all socioeconomic vulnerable patients were identified as such. Moreover, it covers already a measure to combat inequalities in care uptake.

Finally, the use of a probability sample ensures generalisability of our findings to the larger population. Nevertheless, some caution is needed, as the non-response rate of PCPs was relatively high at 73.9%, and the calculated sample weights only allowed adjustment for selective non-response in terms of practice type and region.

## Conclusion and implications

This observational study has shown that practices’ higher level of CCM implementation is associated with an improved patient care process. However, we did not observe significant associations with the outcome QIs. When considering the different elements of the CCM, especially a successful implementation of community linkages and a health information system were related to a better T2D care process. To some extent, the CCM can contribute to improving health equity (i.e. the quintuple aim), by reducing the inequity gap in the T2D care process between socioeconomic vulnerable patients and their counterparts.

Our study has implications for policy, practice and research. The CCM demonstrated its effectiveness in reducing the healthcare inequity gap for patients with T2D. Hence, one potential avenue is to stimulate healthcare practices to improve the quality of care by using CCM in regions with significant socioeconomic inequalities or high concentrations of deprived individuals. To achieve this, initial efforts could encompass training or other innovative methods to steer healthcare organisations. The implementation of the CCM is, however, closely tied to the design of health systems. With equity in mind, policymakers could therefore consider reforms such as integrating financing systems, fostering interdisciplinary collaboration by uniting healthcare workers, and promoting structured, proactive care rather than the prevailing reactive approach. For practitioners, the findings from our study can serve as a basis to reflect on how to better structure their PCPs according to CCM. This involves not only improving the care provided to patients who are already engaged with healthcare services, but also reaching those who do not, or insufficiently, access these services — patients who may benefit even more from enhanced care.

Finally, more research should focus on the interplay between practice organisation and health inequities; our research is only a glimpse of what still can be uncovered. However, it is crucial to recognize that implementing the CCM alone may not be sufficient to fully eliminate healthcare disparities. Addressing the root causes of poverty and deprivation is equally essential. By addressing these structural issues, we can create an environment where the benefits of the CCM can be fully realised.

## Supplementary Information

Supplementary Material 1

## Data Availability

The data that support the findings of this study were used under license for the current study and so are not publicly available.

## Abbreviations

CCM: Chronic Care Model
T2D: type 2 diabetes
ICCC: Innovative Care for Chronic Conditions
eCCM: Expanded Chronic Care Model
MCM: Manage Care Model
PCP: primary care practice
FFS: fee-for-service
IMA: Intermutualistic Agency
LDL-C: low-density lipoprotein cholesterol
ACIC: Assessment of Chronic Illness Care
IR: Increased reimbursement
GLMM: Generalized-linear mixed model
LMM: Linear-mixed model
ICCp: intraclass correlation coefficient for the practice level
PCVp: proportional change in practice-level variance
AOR: adjusted odds ratio
95% CI: 95% confidence interval
-2LL: deviance
df: degrees of freedom
