# Supplementary material for: Navigating diabetes care inequities: an observational study linking chronic care model’s structural elements to process and outcomes of type 2 diabetes care in Belgium
Source: Int J Equity Health. 2025 Jan 20;24:15. doi: 10.1186/s12939-024-02372-4 (PMC11744845; doi:10.1186/s12939-024-02372-4)
Supplement: Supplementary file 1 — Supplementary Material 1 [file 12939_2024_2372_MOESM1_ESM.pdf]

Table of contents

Text A.1 Operationalization of the number of comorbidities variable 2

Text A.2 Calculation of weights 3

Table A.1 Distribution of person-years by process and outcome QIs and covariates for the cohort of T2D patients and the lab data subsample (weighted). 5

Table A.2 Results of the generalized mixed-effects models 0-8 for the association between the ACIC (subscale) scores and the HbA1c process indicator. 6

Table A.3 Results of the generalized mixed-effects models 0-8 for the association between the ACIC (subscale) scores and the LDL-C process indicator. 8

Table A.4 Results of the linear mixed-effects models 0-8 for the association between the ACIC (subscale) scores and yearly average HbA1c values. 10

Table A.5 Results of the linear mixed-effects models 0-8 on the association between the ACIC (subscale) scores and yearly average LDL-C values. 12

Table A.6 Results of the generalized mixed-effects models 1-8 testing the cross-level interactions of the ACIC (subscale) scores with increased reimbursement on the HbA1c process indicator. 14

Table A.7 Results of the generalized mixed-effects models 1-8 testing the cross-level interactions of the ACIC (subscale) scores with increased reimbursement on the LDL-C process indicator. 16

Table A.8 Results of the linear mixed-effects models 1-8 testing the cross-level interactions of the ACIC (subscale) scores with increased reimbursement on yearly average HbA1c values. 18

Table A.9 Results of the linear mixed-effects models 1-8 testing the cross-level interactions of the ACIC (subscale) scores with increased reimbursement on yearly average LDL-C levels. 20

Text A.1 *Operationalization of the number of comorbidities variable*

The number of comorbidities is a continuous variable measuring the number of co-occurring chronic diseases or disease groups that patients have in addition to T2D. These chronic conditions were identified using algorithms that assign chronic conditions to patients based on the minimum of 90 defined daily doses of certain classes of prescribed medication dispensed in public pharmacies, and sometimes also based on the age of the patient [1, 2]. The list of comorbidities comprised the following (groups of) chronic conditions: cardiovascular diseases, thrombosis, chronic obstructive pulmonary disease, asthma, cystic fibrosis, psoriasis, exocrine pancreatic insufficiency, psychosis, Parkinson's disease, epilepsy and neuropathic pain, HIV, hepatitis, multiple sclerosis, organ transplantation, renal failure, thyroid disorders, haemophilia, Alzheimer's disease, and other autoimmune inflammatory conditions (including Crohn's disease, ulcerative colitis, psoriatic arthritis, rheumatoid arthritis). The latter were considered as a single group in the calculations, due to the similarities in medication usage for treatment, not allowing to distinguish them from one another.

Text A.2 *Calculation of weights*

The overall individual case weights for j^{th} patient in practice i is given by

$$w_{ij} = w_{ij,d} * w_{ij,nr}$$

where $w_{ij,d}$ and $w_{ij,nr}$ are respectively the design weight correcting for unequal selection probabilities induced by the disproportionate stratification and the adjustment for non-response [3].

The design weights are the reciprocals of the selection probabilities of patients in our sample[3], so that

$$w_{ij}, d = \frac{1}{\pi_{ij}}$$

whereby π_{ij} reflects the sampling probability of the jth patient of practice i. In a clustered sampling design with stratification at the level of primary care practices, the sampling probability for the jth patient of practice i is

$$\pi_{ij=} \frac{m_{k(i)}}{M_{k(i)}} * \frac{n_{ij}}{N_{ij}}$$

where $m_{k(i)}$ denotes the number of selected primary care practices in stratum k, $M_{k(i)}$ denotes the total number of practices in stratum k, n_{ij} denotes the number of patients of practice i selected in the sample and N_{ij} denotes the total number of patients of practice i. However, as we used a one-stage cluster sampling design, n_{ij} = N_{ij} and thus the formula simplifies so that the design weight is calculated as

$$w_{ij} = \frac{M_{k(i)}}{m_{k(i)}}$$

Because the non-response of primary care practices was relative high – with an overall non-response rate amounting to 73.9% – and strongly selective in terms of practice type – with non-response rates ranging from 5.9% for the multidisciplinary practices with a capitation-based financing system to as high as 86.2% for the monodisciplinary practices with a FFS financing system – an adjustment of the sample weights to compensate for this selective non-response was necessary.

Unfortunately, as no other information than the stratification variables – practice type and region – of the practices that refused to participate in our study was available, the non-response adjustment only corrects the sample in terms of these known characteristics and assumes that within these strata, non-response was random. The non-response weight for patient j of practice i is calculated as [3]

$$w_{ij,nr} = \frac{1}{r_{k(i)}}$$

where $r_{k(i)}$ denotes the response rate of practices belonging to stratum k.

***Table A.1** Distribution of person-years by process and outcome QIs and covariates for the cohort of T2D patients and the lab data subsample (weighted).*

**Level**	**Variable**	**IMA sample**	**lab data subsample**
Patient characteristics		n = 7593	n = 4549
	HbA1c process QI (%)	68.5	
	LDL-C process QI (%)		75.3
	HbA1c outcome QI (mean (SD))		7.04 (1.04)
	LDL-C outcome QI (mean (SD))		82.44 (31.00)
	age (%)		
	40-59 years	25.1	22.2
	60-79 years	56.7	57.7
	80+ years	18.1	20.1
	Male sex (%)	55.6	53.7
	increased reimbursement status (%)	32.9	29.6
	Number of comorbidities (mean (SD))	1.58 (0.95)	1.63 (0.95)
	Number of GPs (mean (SD))	1.30 (0.65)	1.28 (0.64)
	lives alone (%)	31.8	31.3
	died during observation window (%)	2.6	2.5
Practice characteristics		n = 58	n= 58
	ACIC scores (0 – 11) (mean (SD))		
	Total ACIC score	3.65 (1.16)	3.83 (1.13)
	Healthcare Organization	5.98 (1.55)	6.41 (1.52)
	Community linkage	2.38 (1.52)	2.70 (1.50)
	Self-management support	3.72 (1.55)	3.95 (1.42)
	Decision support	3.21 (0.97)	3.21 (0.91)
	Delivery system design	3.20 (1.85)	3.19 (1.84)
	Clinical information system	3.38 (1.94)	3.55 (2.11)
	Practice type (%)		
	monodisciplinary fee-for-service	74.2	75.2
	multidisciplinary fee-for-service	19.3	19.0
	multidisciplinary capitation	6.5	5.8
total person-years		n = 21.939	n = 13.251

Notes: SD = Standard deviation

***Table A.2** Results of the generalized mixed-effects models 0-8 for the association between the ACIC (subscale) scores and the HbA1c process indicator.*

	Model 0		Model 1		Model 2		Model 3		Model 4	
	**exp(b)**	**sig.**	**exp(b)**	**sig.**	**exp(b)**	**sig.**	**exp(b)**	**sig.**	**exp(b)**	**sig.**
**Fixed effects**										
Intercept	6.47	***	4.09	***	12.58	***	4.05	***	7.37	***
Age at baseline										
40-59			Ref.		Ref.		Ref.		Ref.	
60-79			0.7	**	0.7	**	0.7	**	0.69	**
80+			0.24	***	0.24	***	0.24	***	0.24	***
Male sex			0.96		0.96		0.96		0.96	
Increased reimbursement			0.67	***	0.67	***	0.67	***	0.67	***
# comorbidities			2.6	***	2.6	***	2.6	***	2.6	***
# comorbidities (quadratic)			0.85	***	0.85	***	0.85	***	0.85	***
# GPs			1.33	***	1.33	***	1.33	***	1.33	***
# GPs (quadratic)			0.98	**	0.98	**	0.98	**	0.98	**
Living alone			1.23	**	1.23	**	1.23	**	1.23	**
Deceased			0.07	***	0.07	***	0.07	***	0.07	***
Year										
2017			Ref.		Ref.		Ref.		Ref.	
2018			1.07	**	1.07	**	1.07	**	1.07	**
2019			1.22	***	1.22	***	1.22	***	1.22	***
Practice type										
monodiscip. FFS			Ref.		Ref.		Ref.		Ref.	
multidiscip. FFS			0.92		0.38		0.96		0.55	
multidiscip. capittion			1.23		0.13	*	1.18		0.31	
ACIC (subscale) scores										
total ACIC score					2.17	**				
healhcare organization							1.08			
community linkage									1.59	**
self-management support										
decision support										
delivery system design										
clinical information system										
**Random effects**										
Individual variance (intercept)	14.20		14.27		14.27		14.27		14.28	
Practice variance (intercept)	3.43		3.56		2.81		3.54		2.74	
ICC_i	67.88		67.56		70.07		67.65		70.31	
ICC_p	16.40		16.87		13.78		16.76		13.49	
**Model parameters**										
N person-years	22129		22129		22129		22129		22129	
N patients	7608		7608		7608		7608		7608	
N practices	58		58		58		58		58	
Deviance (-2LL)	68406.54		66503.1		66490		66502.76		66489.02	

*Notes: *** p ≤ 0.001; ** p ≤0.01; * p ≤ 0.05; FFS = fee-for-service; ICC_i = intraclass correlation coefficient for the individual level ICC_p = intraclass correlation coefficient for the practice level*

Table A.2 *(continued).*

	Model 5		Model 6		Model 7		Model 8	
	**exp(b)**	**sig.**	**exp(b)**	**sig.**	**exp(b)**	**sig.**	**exp(b)**	**sig.**
**Fixed effects**								
Intercept	7.98	***	5.85	***	12.14	***	7.59	***
Age at baseline								
40-59	Ref.		Ref.		Ref.		Ref.	
60-79	0.7	**	0.7	**	0.7	**	0.7	**
80+	0.24	***	0.24	***	0.24	***	0.24	***
Male sex	0.96		0.96		0.96		0.96	
Increased reimbursement	0.67	***	0.67	***	0.67	***	0.67	***
# comorbidities	2.6	***	2.6	***	2.6	***	2.6	***
# comorbidities (quadratic)	0.85	***	0.85	***	0.85	***	0.85	***
# GPs	1.33	***	1.33	***	1.33	***	1.33	***
# GPs (quadratic)	0.98	**	0.98	**	0.98	**	0.98	**
Living alone	1.23	**	1.23	**	1.23	**	1.23	**
Deceased	0.07	***	0.07	***	0.07	***	0.07	***
Year								
2017	Ref.		Ref.		Ref.		Ref.	
2018	1.07	**	1.07	**	1.07	**	1.07	**
2019	1.22	***	1.22	***	1.22	***	1.22	***
Practice type								
monodiscip. FFS	Ref.		Ref.		Ref.		Ref.	
multidiscip. FFS	0.51		0.63		0.38		0.59	
multidiscip. capitation	0.36		0.63		0.17		0.37	
ACIC (subscale) scores								
total ACIC score								
healhcare organization								
community linkage								
self-management support	1.39							
decision support			1.44					
delivery system design					1.5			
clinical information system							1.39	*
**Random effects**								
Individual variance (intercept)	14.27		14.27		14.27		14.27	
Practice variance (intercept)	3.32		3.36		3.17		3.05	
ICC_i	68.33		68.22		68.84		69.24	
ICC_p	15.92		16.06		15.29		14.80	
**Model parameters**								
N person-years	22129		22129		22129		22129	
N patients	7608		7608		7608		7608	
N practices	58		58		58		58	
Deviance (-2LL)	66499.08		66499.9		66496.66		66494.42	

*Notes: *** p ≤ 0.001; ** p ≤0.01; * p ≤ 0.05; FFS = fee-for-service; ICC_i = intraclass correlation coefficient for the individual level ICC_p = intraclass correlation coefficient for the practice level*

Table A.3 *Results of the generalized mixed-effects models 0-8 for the association between the ACIC (subscale) scores and the LDL-C process indicator.*

	Model 0		Model 1		Model 2		Model 3		Model 4	
	**exp(b)**	**sig.**	**exp(b)**	**sig.**	**exp(b)**	**sig.**	**exp(b)**	**sig.**	**exp(b)**	**sig.**
**Fixed effects**										
Intercept	4.91	***	11.18	***	36.3	***	12.72	***	26.68	***
Age at baseline										
40-59			Ref.		Ref.		Ref.		Ref.	
60-79			1.21		1.21		1.21		1.21	
80+			0.4	***	0.41	***	0.4	***	0.4	***
Male sex			1.14		1.14		1.14		1.14	
Increased reimbursement			1.3	**	1.29	**	1.3	**	1.29	**
# comorbidities			1.24	***	1.24	***	1.24	***	1.24	***
# GPs			1.1	***	1.1	***	1.1	***	1.1	***
Living alone			1.13		1.13		1.13		1.13	
Deceased			0.29	***	0.29	***	0.29	***	0.29	***
Year										
2017			Ref.		Ref.		Ref.		Ref.	
2018			0.77	***	0.77	***	0.77	***	0.77	***
2019			0.62	***	0.62	***	0.62	***	0.62	***
Practice type										
monodiscip. FFS			Ref.		Ref.		Ref.		Ref.	
multidiscip. FFS			0.24	*	0.1	**	0.29		0.13	***
multidiscip. capitation			0.12	**	0.01	***	0.1	***	0.02	***
ACIC (subscale) scores										
total ACIC score					2.07	*				
healhcare organization							1.39			
community linkage									1.75	**
self-management support										
decision support										
delivery system design										
clinical information system										
**Random effects**										
Individual variance (intercept)	8.47		8.60		8.59		8.59		8.60	
Practice variance (intercept)	4.70		3.93		3.40		3.72		2.98	
ICC_i	51.45		54.35		56.24		55.06		57.82	
ICC_p	28.57		24.85		22.23		23.84		20.05	
**Model parameters**										
N person-years	13349		13349		13349		13349		13349	
N patients	4556		4556		4556		4556		4556	
N practices	58		58		58		58		58	
Deviance (-2LL)	43086.48		42440.5		42432.74		42436.08		42425.92	

*Notes: *** $p \leq 0.001$; ** $p \leq 0.01$; * $p \leq 0.05$; FFS = fee-for-service; ICC_i = intraclass correlation coefficient for the individual level ICC_p = intraclass correlation coefficient for the practice level*

***Table A.3** (continued).*

	Model 5		Model 6		Model 7		Model 8	
	exp(b)	sig.	exp(b)	sig.	exp(b)	sig.	exp(b)	sig.
**Fixed effects**								
Intercept	21.2	***	13.95	***	10.19	**	17.08	***
Age at baseline								
40-59	Ref.		Ref.		Ref.		Ref.	
60-79	1.21		1.21		1.21		1.21	
80+	0.4	***	0.4	***	0.4	***	0.4	***
Male sex	1.14		1.14		1.14		1.14	
Increased reimbursement	1.3	**	1.3	**	1.3	**	1.3	**
# comorbidities	1.24	***	1.24	***	1.24	***	1.24	***
# GPs	1.1	***	1.1	***	1.1	***	1.1	***
Living alone	1.13		1.13		1.13		1.13	
Deceased	0.29	***	0.29	***	0.29	***	0.29	***
Year								
2017	Ref.		Ref.		Ref.		Ref.	
2018	0.77	***	0.77	***	0.77	***	0.77	***
2019	0.62	***	0.62	***	0.62	***	0.62	***
Practice type								
monodiscip. FFS	Ref.		Ref.		Ref.		Ref.	
multidiscip. FFS	0.14	*	0.19	*	0.26		0.18	*
multidiscip. capitation	0.04	**	0.08	**	0.15		0.06	**
ACIC (subscale) scores								
total ACIC score								
healhcare organization								
community linkage								
self-management support	1.35							
decision support			1.27					
delivery system design					0.96			
clinical information system							1.21	
**Random effects**								
Individual variance (intercept)	8.60		8.60		8.60		8.60	
Practice variance (intercept)	3.76		3.84		3.93		3.73	
ICC_i	54.96		54.67		54.35		55.04	
ICC_p	24.01		24.41		24.86		23.90	
**Model parameters**								
N person-years	13349		13349		13349		13349	
N patients	4556		4556		4556		4556	
N practices	58		58		58		58	
Deviance (-2LL)	42438.06		42439.42		42440.5		42438.28	

*Notes: *** $p \leq 0.001$; ** $p \leq 0.01$; * $p \leq 0.05$; FFS = fee-for-service; ICC_i = intraclass correlation coefficient for the individual level ICC_p = intraclass correlation coefficient for the practice level*

Table A.4 *Results of the linear mixed-effects models 0-8 for the association between the ACIC (subscale) scores and yearly average HbA1c values.*

	Model 0		Model 1		Model 2		Model 3		Model 4	
	**b (SE)**	**sig.**	**b (SE)**	**sig.**	**b (SE)**	**sig.**	**b (SE)**	**sig.**	**b (SE)**	**sig.**
**Fixed effects**										
Intercept	7.14 (0.04)	***	7.24 (0.06)	***	7.22 (0.09)	***	7.24 (0.06)	***	7.24 (0.07)	***
Age at baseline										
40-59			Ref.		Ref.		Ref.		Ref.	
60-79			-0.22 (0.04)	***	-0.22 (0.04)	***	-0.22 (0.04)	***	-0.22 (0.04)	***
80+			-0.25 (0.05)	***	-0.25 (0.05)	***	-0.25 (0.05)	***	-0.25 (0.05)	***
Male sex			0.06 (0.03)		0.06 (0.03)		0.06 (0.03)		0.06 (0.03)	
Increased reimbursement			0.05 (0.03)		0.05 (0.03)		0.05 (0.03)		0.05 (0.03)	
# comorbidities			-0.08 (0.03)	**	-0.08 (0.03)	**	-0.08 (0.03)	**	-0.08 (0.03)	**
# comorbidities (quadratic)			0.02 (0.01)	*	0.02 (0.01)	*	0.02 (0.01)	*	0.02 (0.01)	*
# GPs			-0.01 (0.01)		-0.01 (0.01)		-0.01 (0.01)		-0.01 (0.01)	
Living alone			-0.01 (0.03)		-0.01 (0.03)		-0.01 (0.03)		-0.01 (0.03)	
Deceased			-0.17 (0.05)	**	-0.17 (0.05)	**	-0.17 (0.05)	**	-0.17 (0.05)	**
Year										
2017			Ref.		Ref.		Ref.		Ref.	
2018			-0.01 (0.01)		-0.01 (0.01)		-0.01 (0.01)		-0.01 (0.01)	
2019			0.06 (0.01)	***	0.06 (0.01)	***	0.06 (0.01)	***	0.06 (0.01)	***
Practice type										
monodiscip. FFS			-0.01 (0.01)		-0.01 (0.01)		-0.01 (0.01)		-0.01 (0.01)	
multidiscip. FFS			-0.00 (0.07)		0.02 (0.09)		-0.00 (0.07)		0.00 (0.08)	
multidiscip. capitation			0.29 (0.08)	***	0.33 (0.16)	*	0.30 (0.09)	***	0.29 (0.11)	**
ACIC (subscale) scores										
total ACIC score					-0.01 (0.04)					
healhcare organization							-0.01 (0.02)			
community linkage									-0.00 (0.02)	
self-management support										
decision support										
delivery system design										
clinical information system										
**Random effects**										
residual variance (intercept)	1.42		1.41		1.41		1.41		1.41	
Individual variance (intercept)	0.06		0.03		0.03		0.03		0.03	
Practice variance (intercept)	0.36		1.41		1.41		1.41		1.41	
ICC_i	33.49		34.37		34.35		34.35		34.35	
ICC_p	2.52		1.19		1.25		1.25		1.25	
**Model parameters**										
N person-years	11183		11183		11183		11183		11183	
N patients	4394		4394		4394		4394		4394	
N practices	57		57		57		57		57	
Deviance (-2LL)	31292.34		31248.38		31252.76		31253.9		31254.12	

*Notes: *** p ≤ 0.001; ** p ≤0.01; * p ≤ 0.05; FFS = fee-for-service; ICC_i = intraclass correlation coefficient for the individual level ICC_p = intraclass correlation coefficient for the practice level*

***Table A.4** (continued).*

	Model 5		Model 6		Model 7		Model 8	
	b (SE)	sig.	b (SE)	sig.	b (SE)	sig.	b (SE)	sig.
**Fixed effects**								
Intercept	7.22 (0.09)	***	7.25 (0.07)	***	7.27 (0.09)	***	7.21 (0.08)	***
Age at baseline								
40-59	Ref.		Ref.		Ref.		Ref.	
60-79	-0.22 (0.04)	***	-0.22 (0.04)	***	-0.22 (0.04)	***	-0.22 (0.04)	***
80+	-0.25 (0.05)	***	-0.25 (0.05)	***	-0.25 (0.05)	***	-0.25 (0.05)	***
Male sex	0.06 (0.03)		0.06 (0.03)		0.06 (0.03)		0.06 (0.03)	
Increased reimbursement	0.05 (0.03)		0.05 (0.03)		0.05 (0.03)		0.05 (0.03)	
# comorbidities	-0.08 (0.03)	**	-0.08 (0.03)	**	-0.08 (0.03)	**	-0.08 (0.03)	**
# comorbidities (quadratic)	0.02 (0.01)	*	0.02 (0.01)	*	0.02 (0.01)	*	0.02 (0.01)	*
# GPs	-0.01 (0.01)		-0.01 (0.01)		-0.01 (0.01)		-0.01 (0.01)	
Lives alone	-0.01 (0.03)		-0.01 (0.03)		-0.01 (0.03)		-0.01 (0.03)	
Deceased	-0.17 (0.05)	**	-0.17 (0.05)	**	-0.17 (0.05)	**	-0.17 (0.05)	**
Year								
2017	Ref.		Ref.		Ref.		Ref.	
2018	-0.01 (0.01)		-0.01 (0.01)		-0.01 (0.01)		-0.01 (0.01)	
2019	0.06 (0.01)	***	0.06 (0.01)	***	0.06 (0.01)	***	0.06 (0.01)	***
Practice type								
monodiscip. FFS	-0.01 (0.01)		-0.01 (0.01)		-0.01 (0.01)		-0.01 (0.01)	
multidiscip. FFS	0.02 (0.09)		-0.00 (0.08)		-0.02 (0.09)		0.02 (0.08)	
multidiscip. capitation	0.33 (0.14)	*	0.28 (0.10)	**	0.23 (0.16)		0.35 (0.11)	**
ACIC (subscale) scores								
total ACIC score								
healhcare organization								
community linkage								
self-management support	-0.01 (0.03)							
decision support			0.00 (0.04)					
delivery system design					0.01 (0.03)			
clinical information system							-0.02 (0.02)	
**Random effects**								
residual variance (intercept)	1.41		1.41		1.41		1.41	
Individual variance (intercept)	0.03		0.03		0.03		0.03	
Practice variance (intercept)	1.41		1.41		1.41		1.41	
ICC_i	34.36		34.36		34.35		34.37	
ICC_p	1.23		1.24		1.25		1.21	
**Model parameters**								
N person-years	11183		11183		11183		11183	
N patients	4394		4394		4394		4394	
N practices	57		57		57		57	
Deviance (-2LL)	31253.36		31253.18		31253.56		31253.82	

*Notes: *** p ≤ 0.001; ** p ≤0.01; * p ≤ 0.05; FFS = fee-for-service; ICC_i = intraclass correlation coefficient for the individual level ICC_p = intraclass correlation coefficient for the practice level*

***Table A.5** Results of the linear mixed-effects models 0-8 on the association between the ACIC (subscale) scores and yearly average LDL-C values.*

	**Model 0**		**Model 1**		**Model 2**		**Model 3**		**Model 4**	
	**b (SE)**	**sig.**	**b (SE)**	**sig.**	**b (SE)**	**sig.**	**b (SE)**	**sig.**	**b (SE)**	**sig.**
**Fixed effects**										
Intercept	85.19 (0.91)	***	101.53 (1.66)	***	98.48 (2.08)	***	101.60 (1.67)	***	100.56 (1.80)	***
Age at baseline										
40-59			Ref.		Ref.		Ref.		Ref.	
60-79			-7.22 (1.14)	***	-7.21 (1.14)	***	-7.22 (1.14)	***	-7.20 (1.14)	***
80+			-0.66 (1.54)		-0.71 (1.54)		-0.67 (1.54)		-0.67 (1.54)	
Male sex			-7.80 (0.92)	***	-7.78 (0.92)	***	-7.80 (0.92)	***	-7.79 (0.92)	***
Increased reimbursement			0.03 (0.91)		0.08 (0.91)		0.02 (0.91)		0.06 (0.91)	
# comorbidities			-6.58 (0.97)	***	-6.58 (0.97)	***	-6.58 (0.97)	***	-6.57 (0.97)	***
# comorbidities (quadratic)			0.89 (0.25)	***	0.89 (0.25)	***	0.89 (0.25)	***	0.89 (0.25)	***
# GPs			1.13 (0.38)	**	1.10 (0.38)	**	1.13 (0.38)	**	1.11 (0.38)	**
Living alone			0.82 (0.91)		0.80 (0.91)		0.82 (0.91)		0.80 (0.91)	
Deceased			-4.27 (1.80)	*	-4.22 (1.80)	*	-4.28 (1.80)	*	-4.26 (1.80)	*
Year										
2017			Ref.		Ref.		Ref.		Ref.	
2018			-4.35 (0.41)	***	-4.35 (0.41)	***	-4.35 (0.41)	***	-4.35 (0.41)	***
2019			-4.72 (0.42)	***	-4.72 (0.42)	***	-4.72 (0.42)	***	-4.72 (0.42)	***
Practice type										
monodiscip. FFS			Ref.		Ref.		Ref.		Ref.	
multidiscip. FFS			0.94 (1.57)		3.66 (1.92)		0.97 (1.61)		1.97 (1.74)	
multidiscip. capitation			6.55 (1.92)	***	13.39 (3.46)	***	6.35 (2.11)	**	8.71 (2.45)	***
ACIC (subscale) scores										
total ACIC score					-2.20 (0.94)	*				
healhcare organization							0.13 (0.56)			
community linkage									-0.75 (0.52)	
self-management support										
decision support										
delivery system design										
clinical information system										
**Random effects**										
residual variance (intercept)	1305.91		1272.37		1272.44		1272.41		1272.45	
Individual variance (intercept)	632.16		584.10		583.56		583.99		583.66	
Practice variance (intercept)	23.09		9.88		8.79		10.57		10.24	
ICC_i	32.23		31.30		31.29		31.28		31.27	
ICC_p	1.18		0.53		0.47		0.57		0.55	
**Model parameters**										
N person-years	9862		9862		9862		9862		9862	
N patients	4192		4192		4192		4192		4192	
N practices	58		58		58		58		58	
Deviance (-2LL)	94508.04		94048.36		94041.22		94047.64		94045.76	

*Notes: *** p ≤ 0.001; ** p ≤0.01; * p ≤ 0.05; FFS = fee-for-service; ICC_i = intraclass correlation coefficient for the individual level ICC_p = intraclass correlation coefficient for the practice level*

***Table A.5** (continued).*

	Model 5		Model 6		Model 7		Model 8	
	b (SE)	sig.	b (SE)	sig.	b (SE)	sig.	b (SE)	sig.
**Fixed effects**								
Intercept	98.97 (2.05)	***	100.74 (1.78)	***	98.64 (2.08)	***	100.00 (1.87)	***
Age at baseline								
40-59	Ref.		Ref.		Ref.		Ref.	
60-79	-7.21 (1.14)	***	-7.21 (1.14)	***	-7.27 (1.14)	***	-7.23 (1.14)	***
80+	-0.65 (1.54)		-0.64 (1.54)		-0.82 (1.54)		-0.72 (1.54)	
Male sex	-7.78 (0.92)	***	-7.81 (0.92)	***	-7.78 (0.92)	***	-7.79 (0.92)	***
Increased reimbursement	0.01 (0.91)		0.04 (0.91)		0.08 (0.91)		0.04 (0.91)	
# comorbidities	-6.58 (0.97)	***	-6.58 (0.97)	***	-6.59 (0.97)	***	-6.59 (0.97)	***
# comorbidities (quadratic)	0.89 (0.25)	***	0.89 (0.25)	***	0.89 (0.25)	***	0.89 (0.25)	***
# GPs	1.11 (0.38)	**	1.12 (0.38)	**	1.13 (0.38)	**	1.11 (0.38)	**
Living alone	0.83 (0.91)		0.80 (0.91)		0.78 (0.91)		0.81 (0.91)	
Deceased	-4.22 (1.80)	*	-4.27 (1.80)	*	-4.26 (1.80)	*	-4.26 (1.80)	*
Year								
2017	Ref.		Ref.		Ref.		Ref.	
2018	-4.35 (0.41)	***	-4.35 (0.41)	***	-4.34 (0.41)	***	-4.35 (0.41)	***
2019	-4.72 (0.42)	***	-4.72 (0.42)	***	-4.72 (0.42)	***	-4.72 (0.42)	***
Practice type								
monodiscip. FFS	Ref.		Ref.		Ref.		Ref.	
multidiscip. FFS	3.46 (1.95)		1.99 (1.80)		3.49 (1.91)		2.20 (1.74)	
multidiscip. capitation	11.98 (3.25)	***	8.27 (2.37)	***	13.09 (3.48)	***	9.71 (2.61)	***
ACIC (subscale) scores								
total ACIC score								
healhcare organization								
community linkage								
self-management support	-1.43 (0.71)	*						
decision support			-1.10 (0.85)					
delivery system design					-1.33 (0.60)	*		
clinical information system							-0.78 (0.43)	
**Random effects**								
person-year-level var (intercept)	1272.52		1272.43		1272.49		1272.41	
Individual-level var (intercept)	584.02		583.63		583.63		583.46	
Practice-level var (intercept)	8.18		10.60		8.76		10.26	
ICC_i	31.32		31.27		31.30		31.27	
ICC_p	0.44		0.57		0.47		0.55	
**Model parameters**								
N person-years	9862		9862		9862		9862	
N patients	4192		4192		4192		4192	
N practices	58		58		58		58	
Deviance (-2LL)	94043.28		94045.18		94042.68		94044.96	

*Notes: *** p ≤ 0.001; ** p ≤0.01; * p ≤ 0.05; FFS = fee-for-service; ICC_i = intraclass correlation coefficient for the individual level ICC_p = intraclass correlation coefficient for the practice level*

Table A.6 *Results of the generalized mixed-effects models 1-8 testing the cross-level interactions of the ACIC (subscale) scores with increased reimbursement on the HbA1c process indicator.*

	**Model 1**		**Model 2**		**Model 3**		**Model 4**	
	**exp(b)**	**sig.**	**exp(b)**	**sig.**	**exp(b)**	**sig.**	**exp(b)**	**sig.**
**Fixed effects**								
Intercept	3.56	**	10.39	***	3.51	**	6.73	***
Age at baseline								
40-59	Ref.		Ref.		Ref.		Ref.	
60-79	0.7	**	0.7	**	0.7	**	0.7	**
80+	0.25	***	0.25	***	0.25	***	0.25	***
Male sex	0.99		0.99		0.99		0.99	
Increased reimbursement	0.65	*	0.71		0.65	*	0.67	*
# comorbidities	2.59	***	2.59	***	2.59	***	2.59	***
# comorbidities (quadratic)	0.85	***	0.85	***	0.85	***	0.85	***
# GPs	1.34	***	1.34	***	1.34	***	1.34	***
# GPs (quadratic)	0.98	**	0.98	**	0.98	**	0.98	**
Living alone	1.2	*	1.2	*	1.2	*	1.2	*
Deceased	0.07	***	0.07	***	0.07	***	0.07	***
Year								
2017	Ref.		Ref.		Ref.		Ref.	
2018	1.08	**	1.08	**	1.08	**	1.08	**
2019	1.22	***	1.22	***	1.22	***	1.22	***
Practice type								
monodiscip. FFS	Ref.		Ref.		Ref.		Ref.	
multidiscip. FFS	1.13		0.45		1.2		0.61	
multidiscip. capitation	1.61		0.16		1.56		0.36	
ACIC (subscale) scores (main effect)								
total ACIC score			1.77					
healhcare organization					1.04			
community linkage							1.46	
self-management support								
decision support								
delivery system design								
clinical information system								
Increased reimbursement *								
total ACIC score			1.36	*				
healthcare organization					1.07			
community linkage							1.16	*
self-management support								
decision support								
delivery system design								
clinical information system								
**Random effects**								
Individual variance (intercept)	14.53		14.52		14.53		14.53	
Practice variance (intercept)	4.35		3.36		4.34		3.23	
Practice variance (increased reimbursement)	1.58		1.28		1.56		1.49	
practice covariance (intercept, increased reimbursement)	-1.13		-0.80		-1.15		-0.79	
**Model parameters**								
N person-years	22129		22129		22129		22129	
N patients	7608		7608		7608		7608	
N practices	58		58		58		58	
Deviance (-2LL)	66364.96		66346		66386.02		66350.54	

*Notes: *** p ≤ 0.001; ** p ≤0.01; * p ≤ 0.05; FFS = fee-for-service*

Table A.6 *(continued).*

	Model 5		Model 6		Model 7		Model 8	
	exp(b)	sig.	exp(b)	sig.	exp(b)	sig.	exp(b)	sig.
**Fixed effects**								
Intercept	6.59	**	5.07	**	9.67	**	6.57	***
Age at baseline								
40-59	Ref.		Ref.		Ref.		Ref.	
60-79	0.7	**	0.7	**	0.7	**	0.7	**
80+	0.25	***	0.25	***	0.25	***	0.25	***
Male sex	0.99		0.99		0.99		0.99	
Increased reimbursement	0.71		0.69	*	0.75		0.69	
# comorbidities	2.59	***	2.59	***	2.59	***	2.59	***
# comorbidities (quadratic)	0.85	***	0.85	***	0.85	***	0.85	***
# GPs	1.34	***	1.34	***	1.34	***	1.33	***
# GPs (quadratic)	0.98	**	0.98	**	0.98	**	0.98	**
Living alone	1.2	*	1.2	*	1.2	**	1.2	*
Deceased	0.07	***	0.07	***	0.07	***	0.07	***
Year								
2017	Ref.		Ref.		Ref.		Ref.	
2018	1.08	**	1.08	**	1.08	**	1.08	**
2019	1.22	***	1.22	***	1.23	***	1.22	***
Practice type								
monodiscip. FFS	Ref.		Ref.		Ref.		Ref.	
multidiscip. FFS	0.61		0.75		0.46		0.67	
multidiscip. capitation	0.45		0.74		0.21		0.45	
ACIC (subscale) scores (main effect)								
total ACIC score								
healthcare organization								
community linkage								
self-management support	1.21							
decision support			1.16					
delivery system design					1.3			
clinical information system							1.28	
Increased reimbursement *								
total ACIC score								
healthcare organization								
community linkage								
self-management support	1.25	*						
decision support			1.54	*				
delivery system design					1.26	**		
clinical information system							1.14	
**Random effects**								
Individual variance (intercept)	14.51		14.52		14.52		14.53	
Practice variance (intercept)	3.87		4.00		3.77		3.60	
Practice variance (increased reimbursement)	1.31		1.27		1.15		1.46	
practice covariance (intercept, increased reimbursement)	-0.82		-0.94		-0.83		-0.83	
**Model parameters**								
N person-years	22129		22129		22129		22129	
N patients	7608		7608		7608		7608	
N practices	58		58		58		58	
Deviance (-2LL)	66355.1		66353.58		66347.44		66375.46	

*Notes: *** p ≤ 0.001; ** p ≤0.01; * p ≤ 0.05; FFS = fee-for-service*

Table A.7 *Results of the generalized mixed-effects models 1-8 testing the cross-level interactions of the ACIC (subscale) scores with increased reimbursement on the LDL-C process indicator.*

	**Model 1**		**Model 2**		**Model 3**		**Model 4**	
	**exp(b)**	**sig.**	**exp(b)**	**sig.**	**exp(b)**	**sig.**	**exp(b)**	**sig.**
**Fixed effects**								
Intercept	12.2	***	42.76	***	13.71	***	29.78	***
Age at baseline								
40-59	Ref.		Ref.		Ref.		Ref.	
60-79	1.24		1.24		1.23		1.24	
80+	0.42	***	0.42	***	0.42	***	0.42	***
Male sex	1.12		1.12		1.12		1.12	
Increased reimbursement	1.26		1.14		1.22		1.21	
# comorbidities	1.24	***	1.24	***	1.24	***	1.24	***
# GPs	1.09	***	1.09	***	1.09	***	1.09	***
Living alone	1.17		1.17		1.17		1.18	
Deceased	0.3	***	0.3	***	0.3	***	0.3	***
Year								
2017	Ref.		Ref.		Ref.		Ref.	
2018	0.76	***	0.76	***	0.76	***	0.76	***
2019	0.61	***	0.61	***	0.61	***	0.61	***
Practice type								
monodiscip. FFS								
multidiscip. FFS	0.23	*	0.1	**	0.28		0.12	***
multidiscip. capitation	0.12	**	0.01	***	0.09	***	0.02	***
ACIC (subscale) scores								
total ACIC score			2.35	*				
healhcare organization					1.48			
community linkage							1.85	**
self-management support								
decision support								
delivery system design								
clinical information system								
Increased reimbursement *								
total ACIC score			0.77					
healthcare organization					0.85			
community linkage							0.88	
self-management support								
decision support								
delivery system design								
clinical information system								
**Random effects**								
Individual variance (intercept)	9.07		9.06		9.06		9.07	
Practice variance (intercept)	4.45		3.65		4.15		3.37	
Practice variance (increased reimbursement)	2.32		2.09		2.25		2.23	
practice covariance (intercept, increased reimbursement)	-0.83		-0.48		-0.68		-0.72	
**Model parameters**								
N person-years	13349		13349		13349		13349	
N patients	4556		4556		4556		4556	
N practices	58		58		58		58	
Deviance (-2LL)	42203.16		42191.9		42197.64		42187.24	

*Notes: *** $p \leq 0.001$; ** $p \leq 0.01$; * $p \leq 0.05$; FFS = fee-for-service*

Table A.7 *(continued).*

	Model 5 exp(b)	sig.	Model 6 exp(b)	sig.	Model 7 exp(b)	sig.	Model 8 exp(b)	sig.
**Fixed effects**								
Intercept	24.36	***	15.19	***	11.87	**	19.64	***
Age at baseline								
40-59	Ref.		Ref.		Ref.		Ref.	
60-79	1.24		1.24		1.24		1.24	
80+	0.42	***	0.42	***	0.42	***	0.42	***
Male sex	1.12		1.12		1.12		1.12	
Increased reimbursement	1.18		1.23		1.16		1.14	
# comorbidities	1.24	***	1.24	***	1.24	***	1.24	***
# GPs	1.09	***	1.09	***	1.09	***	1.09	***
Living alone	1.17		1.17		1.17		1.17	
Deceased	0.3	***	0.3	***	0.3	***	0.3	***
Year								
2017	Ref.		Ref.		Ref.		Ref.	
2018	0.76	***	0.76	***	0.76	***	0.76	***
2019	0.61	***	0.61	***	0.61	***	0.61	***
Practice type								
monodiscip. FFS								
multidiscip. FFS	0.13	*	0.18	*	0.24		0.18	*
multidiscip. capitation	0.04	**	0.08	**	0.13		0.05	**
ACIC (subscale) scores (main effect)								
total ACIC score								
healthcare organization								
community linkage								
self-management support	1.46							
decision support			1.4					
delivery system design					1.03			
clinical information system							1.29	
Increased reimbursement *								
total ACIC score								
healthcare organization								
community linkage								
self-management support	0.85							
decision support			0.75					
delivery system design					0.86			
clinical information system							0.86	
**Random effects**								
residual variance (intercept)								
Individual variance (intercept)	9.07		9.07		9.07		9.07	
Practice variance (intercept)	4.20		4.31		4.44		4.08	
Practice variance (increased reimbursement)	2.17		2.13		2.13		2.10	
practice covariance (intercept, increased reimbursement)	-0.74		-0.72		-0.78		-0.60	
**Model parameters**								
N person-years	13349		13349		13349		13349	
N patients	4556		4556		4556		4556	
N practices	58		58		58		58	
Deviance (-2LL)	42198.94		42200.3		42200.62		42197.84	

*Notes: *** p ≤ 0.001; ** p ≤0.01; * p ≤ 0.05; FFS = fee-for-service*

Table A.8 *Results of the linear mixed-effects models 1-8 testing the cross-level interactions of the ACIC (subscale) scores with increased reimbursement on yearly average HbA1c values.*

	Model 1 b (SE)	sig.	Model 2 b (SE)	sig.	Model 3 b (SE)	sig.	Model 4 b (SE)	sig.
**Fixed effects**								
Intercept	7.23 (0.06)	***	7.20 (0.09)	***	7.23 (0.06)	***	7.23 (0.07)	***
Age at baseline								
40-59	Ref.		Ref.		Ref.		Ref.	
60-79	-0.22 (0.04)	***	-0.22 (0.04)	***	-0.22 (0.04)	***	-0.22 (0.04)	***
80+	-0.24 (0.05)	***	-0.24 (0.05)	***	-0.24 (0.05)	***	-0.24 (0.05)	***
Male sex	0.06 (0.03)		0.06 (0.03)		0.06 (0.03)		0.06 (0.03)	
Increased reimbursement	0.06 (0.03)		0.06 (0.04)		0.05 (0.03)		0.06 (0.03)	
# comorbidities	-0.08 (0.03)	**	-0.08 (0.03)	**	-0.08 (0.03)	**	-0.08 (0.03)	**
# comorbidities (quadratic)	0.02 (0.01)	*	0.02 (0.01)	*	0.02 (0.01)	*	0.02 (0.01)	*
# GPs	-0.01 (0.01)		-0.01 (0.01)		-0.01 (0.01)		-0.01 (0.01)	
Lives alone	-0.01 (0.03)		-0.01 (0.03)		-0.01 (0.03)		-0.01 (0.03)	
Deceased	-0.17 (0.05)	**	-0.16 (0.05)	**	-0.16 (0.05)	**	-0.17 (0.05)	**
Year								
2017	Ref.		Ref.		Ref.		Ref.	
2018	-0.01 (0.01)		-0.01 (0.01)		-0.01 (0.01)		-0.01 (0.01)	
2019	0.06 (0.01)	***	0.06 (0.01)	***	0.06 (0.01)	***	0.06 (0.01)	***
Practice type								
monodiscip. FFS								
multidiscip. FFS	0.00 (0.07)		0.02 (0.09)		-0.00 (0.07)		0.00 (0.08)	
multidiscip. capitation	0.29 (0.08)	***	0.35 (0.16)	*	0.29 (0.09)	***	0.30 (0.11)	**
ACIC (subscale) scores								
total ACIC score			-0.03 (0.04)					
healhcare organization					-0.02 (0.03)			
community linkage							-0.01 (0.02)	
self-management support								
decision support								
delivery system design								
clinical information system								
Increased reimbursement *								
total ACIC score			0.01 (0.03)					
healthcare organization					0.04 (0.02)			
community linkage							0.01 (0.02)	
self-management support								
decision support								
delivery system design								
clinical information system								
**Random effects**								
residual variance (intercept)	1.41		1.41		1.41		1.41	
Individual variance (intercept)	0.75		0.75		0.75		0.75	
Practice variance (intercept)	0.03		0.03		0.03		0.03	
Practice variance (increased reimbursement)	0.01		0.01		0		0.01	
practice covariance (intercept, increased reimbursement)	-0.01		-0.01		0		-0.01	
**Model parameters**								
N person-years	11183		11183		11183		11183	
N patients	4394		4394		4394		4394	
N practices	57		57		57		57	
Deviance (-2LL)	31246.2		31255.62		31254.88		31257.66	

*Notes: *** p ≤ 0.001; ** p ≤0.01; * p ≤ 0.05; FFS = fee-for-service*

Table A.8 *(continued).*

	Model 5 b (SE)	sig.	Model 6 b (SE)	sig.	Model 7 b (SE)	sig.	Model 8 b (SE)	sig.
**Fixed effects**								
Intercept	7.21 (0.09)	***	7.24 (0.07)	***	7.25 (0.09)	***	7.18 (0.08)	***
Age at baseline								
40-59	Ref.		Ref.		Ref.		Ref.	
60-79	-0.22 (0.04)	***	-0.21 (0.04)	***	-0.22 (0.04)	***	-0.22 (0.04)	***
80+	-0.24 (0.05)	***	-0.24 (0.05)	***	-0.24 (0.05)	***	-0.24 (0.05)	***
Male sex	0.06 (0.03)		0.06 (0.03)		0.06 (0.03)		0.06 (0.03)	
Increased reimbursement	0.06 (0.03)		0.06 (0.04)		0.06 (0.04)		0.05 (0.04)	
# comorbidities	-0.08 (0.03)	**	-0.08 (0.03)	**	-0.08 (0.03)	**	-0.08 (0.03)	**
# comorbidities (quadratic)	0.02 (0.01)	*	0.02 (0.01)	*	0.02 (0.01)	*	0.02 (0.01)	*
# GPs	-0.01 (0.01)		-0.01 (0.01)		-0.01 (0.01)		-0.01 (0.01)	
Lives alone	-0.01 (0.03)		-0.01 (0.03)		-0.01 (0.03)		-0.01 (0.03)	
Deceased	-0.16 (0.05)	**	-0.17 (0.05)	**	-0.16 (0.05)	**	-0.17 (0.05)	**
Year								
2017	Ref.		Ref.		Ref.		Ref.	
2018	-0.01 (0.01)		-0.01 (0.01)		-0.01 (0.01)		-0.01 (0.01)	
2019	0.06 (0.01)	***	0.06 (0.01)	***	0.06 (0.01)	***	0.06 (0.01)	***
Practice type								
monodiscip. FFS								
multidiscip. FFS	0.02 (0.09)		-0.00 (0.08)		-0.02 (0.09)		0.04 (0.08)	
multidiscip. capitation	0.33 (0.14)	*	0.29 (0.10)	**	0.24 (0.16)		0.42 (0.11)	***
ACIC (subscale) scores								
total ACIC score								
healthcare organization								
community linkage								
self-management support	-0.02 (0.03)							
decision support			0.00 (0.04)					
delivery system design					0.01 (0.03)			
clinical information system							-0.02 (0.02)	
Increased reimbursement *								
total ACIC score								
healthcare organization								
community linkage								
self-management support	0.02 (0.02)							
decision support			-0.00 (0.03)					
delivery system design					0.01 (0.02)			
clinical information system							-0.01 (0.02)	
**Random effects**								
residual variance (intercept)	1.41		1.41		1.41		1.41	
Individual variance (intercept)	0.75		0.75		0.75		0.75	
Practice variance (intercept)	0.03		0.03		0.03		0.03	
Practice variance (increased reimbursement)	0.01		0.01		0.01		0.01	
practice covariance (intercept, increased reimbursement)	-0.01		-0.01		-0.01		-0.01	
**Model parameters**								
N person-years	11183		11183		11183		11183	
N patients	4394		4394		4394		4394	
N practices	57		57		57		57	
Deviance (-2LL)	31256.2		31255.9		31257.56		31257.12	

*Notes: *** p ≤ 0.001; ** p ≤0.01; * p ≤ 0.05; FFS = fee-for-service*

Table A.9 *Results of the linear mixed-effects models 1-8 testing the cross-level interactions of the ACIC (subscale) scores with increased reimbursement on yearly average LDL-C levels.*

	**Model 1**		**Model 2**		**Model 3**		**Model 4**	
	**b (SE)**	**sig.**	**b (SE)**	**sig.**	**b (SE)**	**sig.**	**b (SE)**	**sig.**
**Fixed effects**								
Intercept	101.48 (1.67)	***	97.87 (2.08)	***	101.57 (1.70)	***	100.51 (1.81)	***
Age at baseline								
40-59	Ref.		Ref.		Ref.		Ref.	
60-79	-7.15 (1.14)	***	-7.15 (1.14)	***	-7.19 (1.14)	***	-7.13 (1.14)	***
80+	-0.62 (1.54)		-0.72 (1.54)		-0.61 (1.54)		-0.64 (1.54)	
Male sex	-7.77 (0.92)	***	-7.76 (0.92)	***	-7.77 (0.92)	***	-7.76 (0.92)	***
Increased reimbursement	-0.02 (1.12)		0.28 (1.14)		-0.04 (1.08)		0.07 (1.14)	
# comorbidities	-6.59 (0.97)	***	-6.59 (0.97)	***	-6.58 (0.97)	***	-6.58 (0.97)	***
# comorbidities (quadratic)	0.90 (0.25)	***	0.91 (0.25)	***	0.90 (0.25)	***	0.90 (0.25)	***
# GPs	1.13 (0.38)	**	1.11 (0.38)	**	1.14 (0.38)	**	1.11 (0.38)	**
Living alone	0.81 (0.91)		0.77 (0.91)		0.81 (0.91)		0.79 (0.91)	
Deceased	-4.13 (1.81)	*	-4.01 (1.81)	*	-4.06 (1.81)	*	-4.10 (1.81)	*
Year								
2017	Ref.		Ref.		Ref.		Ref.	
2018	-4.35 (0.41)	***	-4.35 (0.41)	***	-4.35 (0.41)	***	-4.35 (0.41)	***
2019	-4.70 (0.42)	***	-4.70 (0.42)	***	-4.71 (0.42)	***	-4.70 (0.42)	***
Practice type								
monodiscip. FFS								
multidiscip. FFS	0.87 (1.57)		4.02 (1.91)	*	0.94 (1.63)		1.87 (1.75)	
multidiscip. capitation	6.41 (1.92)	***	14.26 (3.50)	***	5.84 (2.12)	**	8.53 (2.48)	***
ACIC (subscale) scores								
total ACIC score			-2.56 (0.96)	**				
healthcare organization					-0.19 (0.63)			
community linkage							-0.73 (0.56)	
self-management support								
decision support								
delivery system design								
clinical information system								
Increased reimbursement *								
total ACIC score			0.29 (0.83)					
healthcare organization					1.12 (0.76)			
community linkage							0.02 (0.67)	
self-management support								
decision support								
delivery system design								
clinical information system								
**Random effects**								
residual variance (intercept)	1271.99		1272.05		1272.12		1272.12	
Individual variance (intercept)	581.34		580.68		581.26		580.81	
Practice variance (intercept)	11.73		8		13.07		11.65	
Practice variance (increased reimbursement)	13.61		13.51		10.53		14.7	
practice covariance (intercept, increased reimbursement)	-5.38		0.19		-5.06		-4.53	
**Model parameters**								
N person-years	9862		9862		9862		9862	
N patients	4192		4192		4192		4192	
N practices	58		58		58		58	
Deviance (-2LL)	94045.7		94036.64		94041.54		94042.26	

*Notes: *** p ≤ 0.001; ** p ≤0.01; * p ≤ 0.05; FFS = fee-for-service*

Table A.9 *(continued).*

	Model 5		Model 6		Model 7		Model 8	
	**b (SE)**	**sig.**	**b (SE)**	**sig.**	**b (SE)**	**sig.**	**b (SE)**	**sig.**
**Fixed effects**								
Intercept	98.42 (2.04)	***	100.61 (1.80)	***	98.46 (1.67)	***	99.89 (1.88)	***
Age at baseline								
40-59	Ref.		Ref.		Ref.		Ref.	
60-79	-7.15 (1.14)	***	-7.14 (1.14)	***	-7.15 (1.14)	***	-7.16 (1.14)	***
80+	-0.63 (1.54)		-0.60 (1.54)		-0.91 (1.54)		-0.70 (1.54)	
Male sex	-7.75 (0.92)	***	-7.78 (0.92)	***	-7.75 (0.92)	***	-7.76 (0.92)	***
Increased reimbursement	0.20 (1.14)		0.11 (1.12)		0.11 (1.16)		0.04 (1.18)	
# comorbidities	-6.60 (0.97)	***	-6.60 (0.97)	***	-6.56 (0.97)	***	-6.59 (0.97)	***
# comorbidities (quadratic)	0.91 (0.25)	***	0.90 (0.25)	***	0.89 (0.25)	***	0.90 (0.25)	***
# GPs	1.12 (0.38)	**	1.13 (0.38)	**	1.15 (0.38)	**	1.12 (0.38)	**
Living alone	0.81 (0.91)		0.79 (0.91)		0.63 (0.91)		0.79 (0.91)	
Deceased	-4.01 (1.81)	*	-4.08 (1.81)	*	-4.07 (1.81)	*	-4.09 (1.81)	*
Year								
2017	Ref.		Ref.		Ref.		Ref.	
2018	-4.34 (0.41)	***	-4.34 (0.41)	***	-4.34 (0.41)	***	-4.35 (0.41)	***
2019	-4.70 (0.42)	***	-4.70 (0.42)	***	-4.69 (0.42)	***	-4.70 (0.42)	***
Practice type								
monodiscip. FFS								
multidiscip. FFS	3.79 (1.94)		1.96 (1.81)		3.54 (1.34)	**	2.14 (1.74)	
multidiscip. capitation	12.66 (3.30)	***	8.07 (2.39)	***	12.63 (2.51)	***	9.67 (2.65)	***
ACIC (subscale) scores								
total ACIC score								
healthcare organization								
community linkage								
self-management support	-1.71 (0.74)	*						
decision support			-1.37 (0.93)					
delivery system design					-1.10 (0.42)	**		
clinical information system							-0.76 (0.45)	
Increased reimbursement *								
total ACIC score								
healthcare organization								
community linkage								
self-management support	0.34 (0.65)							
decision support			0.76 (1.09)					
delivery system design					-0.14 (0.51)			
clinical information system							-0.07 (0.50)	
**Random effects**								
person-year variance (intercept)	1272.19		1271.86		1271.76		1272.14	
Individual variance (intercept)	581.2		581.11		586.91		580.47	
Practice variance (intercept)	7.57		11.99		0		11.01	
Practice variance (increased reimbursement)	13.42		13.43		14.66		15.07	
practice covariance (intercept, increased reimbursement)	-0.66		-4.23		0		-3.47	
**Model parameters**								
N person-years	9862		9862		9862		9862	
N patients	4192		4192		4192		4192	
N practices	58		58		58		58	
Deviance (-2LL)	94039.22		94040.06		94048.18		94042.04	

*Notes: *** p ≤ 0.001; ** p ≤0.01; * p ≤ 0.05; FFS = fee-for-service*

References

1. Berete F, Demarest S, Charafeddine R, Bruyere O, Van der Heyden J. Comparing health insurance data and health interview survey data for ascertaining chronic disease prevalence in Belgium. Arch Public Health. 2020;78(1):120.

2. IMA-AIM. Enchantillon permanente steekproef: EPS R13 – FLAGS Release 20190201 NL 2019. Available from: https://ima-aim.be/IMG/pdf/eps_r13_-_flags_release_20190201_nl_-_vs2.pdf.

3. Kish L. Weighting for unequal Pi. Journal of Official Statistics. 1992;8(2):183.
